# Parametric Design of Hip Implant With Gradient Porous Structure

**DOI:** 10.3389/fbioe.2022.850184

**Published:** 2022-05-16

**Authors:** Xiangsheng Gao, Yuhang Zhao, Min Wang, Ziyu Liu, Chaozong Liu

**Affiliations:** ^1^ Beijing Key Laboratory of Advanced Manufacturing Technology, Faculty of Materials and Manufacturing, Beijing University of Technology, Beijing, China; ^2^ Division of Surgery and Interventional Science, University College London, Royal National Orthopaedic Hospital, London, United Kingdom; ^3^ School of Engineering Medicine, Beihang University, Beijing, China

**Keywords:** hip implant, stress-shielding, kriging algorithm, gradient porous structure, parametric design

## Abstract

Patients who has been implanted with hip implant usually undergo revision surgery. The reason is that high stiff implants would cause non-physiological distribution loadings, which is also known as stress shielding, and finally lead to bone loss and aseptic loosening. Titanium implants are widely used in human bone tissues; however, the subsequent elastic modulus mismatch problem has become increasingly serious, and can lead to stress-shielding effects. This study aimed to develop a parametric design methodology of porous titanium alloy hip implant with gradient elastic modulus, and mitigate the stress-shielding effect. Four independent adjustable dimensions of the porous structure were parametrically designed, and the Kriging algorithm was used to establish the mapping relationship between the four adjustable dimensions and the porosity, surface-to-volume ratio, and elastic modulus. Moreover, the equivalent stress on the surface of the femur was optimized by response surface methodology, and the optimal gradient elastic modulus of the implant was obtained. Finally, through the Kriging approximation model and optimization results of the finite element method, the dimensions of each segment of the porous structure that could effectively mitigate the stress-shielding effect were determined. Experimental results demonstrated that the parameterized design method of the porous implant with gradient elastic modulus proposed in this study increased the strain value on the femoral surface by 17.1% on average. Consequently, the stress-shielding effect of the femoral tissue induced by the titanium alloy implant was effectively mitigated.

## Graphical Abstract

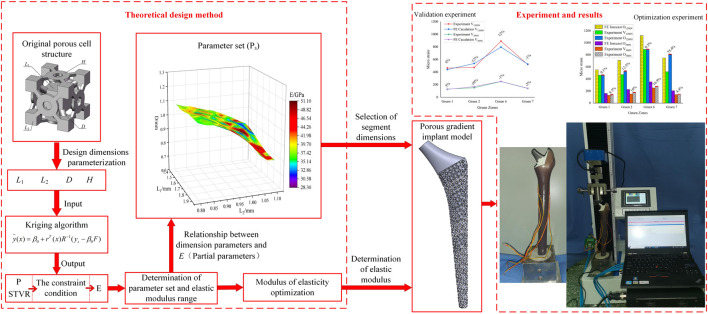

Highlights

➢ Propose a Kriging-based parametric design method for gradient porous structure.
➢ Optimize the elastic modulus based on the response surface methodology to adjust the stress distribution of femur.
➢ Develop an implant with gradient porous structure to mitigate the stress-shielding effect and verified by experiments.

## Introduction

Patients who has been implanted with hip implant may suffer secondary injuries after periprosthetic fracture or feeling severe painfulness. One of the reasons is that high stiff implants would cause non-physiological distribution loadings, which is also known as stress shielding, and finally lead to bone loss and aseptic loosening (Naghavi et al., 2019).

However, inserting a stiff implant into human body would result in non-physiological distribution loadings (Ahmed et al., 2020). In that case, it would cause a decrease of the periprosthetic bone strain (Huiskes et al., 1992) and then lead to bone loss and aseptic loosening (Xu and Robinson, 2008; Jeyapalina et al., 2014). Managing stress distribution would one of the main issue for implants, and the design should provide suitable strain-related stimulus for altering bone mass by bone remodeling (bone resorption or bone apposition) (Prendergast and Taylor, 1994).

In recent years, cellular metallic structures are of particular interest in orthopedic implant applications, since they can be effectively used for the replacement of broken or damaged bones (Abate et al., 2021). In all metal implant materials that can be designed for cell structure, titanium alloys are promising materials used as bone implants due to their unique properties such as high specific strength, excellent biocompatibility, and corrosion resistance (Geetha et al., 2009; Niinomi et al., 2012). Nevertheless, the elastic modulus of human bone (<30 GPa) is much lower than that of solid titanium (∼110 GPa) (Abdel-Hady Gepreel and Niinomi, 2013). The mismatch of the elastic modulus between bone tissues and solid implants causes stress shielding that can weaken the bone and stop bone growth. Previous studies have reported that this stress can be reduced by implants with porous structure, mainly by adjusting their pore size and porosity (Gibson, 2005), (Honda et al., 2013). For example, trabecular metal with a porous structure has been found in several studies to show good results in revision arthroplasty for severe acetabular bone loss (Christie, 2002; Flecher, Sporer, and Paprosky, 2008; Davies et al., 2011). Such porous structures have been proven to provide a firm fixation of the implant (Nazir et al., 2019), since they can not only reduce the elastic modulus of the titanium alloy, but also facilitate bone osseointegration (Takemoto et al., 2005; Wiria et al., 2010; Torres-Sanchez et al., 2017) and in-growth (Babaie and Bhaduri, 2018).

More specifically, the elastic modulus of a titanium alloy can be adjusted by the design of the porous structure, thereby mitigating the stress shielding effect. The elastic modulus of an implant can be custom-defined, and by controlling the porosity, the implant can obtain mechanical properties and structure similar to those of bone (Yan et al., 2015). For example, Lee et al. (2014) investigated the effect of space scaffolding on the structure and mechanical properties of porous titanium, and they concluded that porosity determines the modulus of elasticity. Mullen (Mullen et al., 2009) constructed a honeycomb titanium structure based on octahedral cells and Arabnejad (Arabnejad et al., 2016) developed two high-strength tensile dominant topologies; both exhibited the potential of such structures for orthopaedic implants. Based on newly-designed honeycomb structures and five different existing honeycomb structures, Abate et al. (2020) discussed the optimization effects of cell size, lattice topology, porosity, and honeycomb structure on mechanical properties. Their results demonstrated that the optimized honeycomb structures had much lower stress and deformation. Xu et al. (2021) investigated six composite lattice structures with different support radii consisting of simple cubic, body-centered cubic (BCC), and edge-centered cubic cells. Luxner et al. (2007) and Han et al. (2017) also suggested that BCC structures can have relatively higher porosity controllability and better mechanical properties than other structures. Among these structures, the BCC structure was found more suitable for parametric design. The excellent properties of the BCC structure had also been demonstrated experimentally by Cuadrado (Cuadrado et al., 2017).

Previous studies have assessed the actual performance of implants using porous structures within the femur. For instance, in order to reduce the stiffness and allow the inward growth of bone tissue, Jetté et al. (2018) proposed a hip implant design characterized by a porous structure based on a diamond cubic lattice. Alkhatib et al. (2019) developed finite element (FE) models of titanium alloy porous implants and effective porous implants, and verified that porosity affects the stress-shielding effect. Functional gradient materials (FGM) have gradually become the focus of the implant structure research, providing a new method for implant structure design (Moussa et al., 2020). To minimize the stress shielding effect and extend the life of implants, Sun et al. (2018) developed a FE model for bone implants and provided a general approach for designing patient-specific implants with a gradient modulus distribution. Oshkour et al. (2013) developed a functional gradient hip implant to reduce the stress shielding effect and improve the total hip replacement performance. Limmahakhun et al. (2017) assessed the possibility of using graded porous Co-Cr alloys in implants. A comparison of four shapes of femoral stems demonstrated that round implants undergo less deformation and have lower von Mises stresses (Chethan et al., 2019). In general, implants with a functional gradient structure similar to that of bone tissue are ideal for achieving the required mechanical and biological properties (Leong et al., 2008; Han et al., 2019; Chen H. et al., 2020).

In the design of hip implants with porous structure, the design methods are divided into parametric and non-parametric methods based on whether the porous structure is generated by an algorithm. In non-parametric design, most of the structures were similar, and only minor changes could be made (Chen H. et al., 2020). In parametric design, different methods were mainly used to calculate and predict certain characteristic quantities after parameterization. For instance, Ahmadi et al. (2014) proposed an analytical solution to predict the elastic modulus, Poisson’s ratio, elastic buckling limit, and yield stress of a diamond cell structure based on some specific parameters. Shi et al. (2019) parametrically designed the porous structures based on three-dimensional periodic miniaturized surface (TPMS), which have a good osseointegration effect. However, no parametric optimization of porous structures was conducted in these researches. In terms of the hip implant design, the stress distribution is uneven, the elastic modulus of implant should be adjusted with the stress distribution, so the gradient porous structures should be used in the implant design. Recently, there is a lack of effective methods and corresponding experimental analysis for the femur implant design with gradient modulus.

At present, the Kriging approximation model is widely used in the engineering field. For example, Gao et al. (2013) proposed a Kriging approximation model that can quickly and effectively estimate the dynamic characteristics of the machine tool in order to study the changes of the dynamic characteristics of the machine tool in the manufacturing space. However, it has not been fully developed and applied in the field of biomedical engineering. Therefore, a new parametric design method was proposed to mitigate the stress-shielding effect by optimizing the porous structure of gradient implants using the Kriging algorithm and FE method based on the target elastic modulus on the femoral surface in this study. The contents of this paper are arranged as follows. In Section 2, the development of the FE model of the hip implant was discussed and validation experiments were carried out; in Section 3, a Kriging model was developed to express the relationship between adjustable dimensions and STVR, porosity and elastic modulus, and from these relationships the adjustable dimensions set and the range of elastic modulus for subsequent optimization were derived. Through the FE method, optimization of the elastic modulus was carried out based on the constraint range of elastic modulus, and the optimal porous structure dimensions were determined based on the optimized elastic modulus and the adjustable dimension parameter set; in Section 4, the effects of the optimized design were analyzed and experimentally verified, and finally, in Section 5, the content of this research was summarized.

## Modeling of Femur Bone/Implant Interaction

### FE Modeling

**FIGURE 1:**
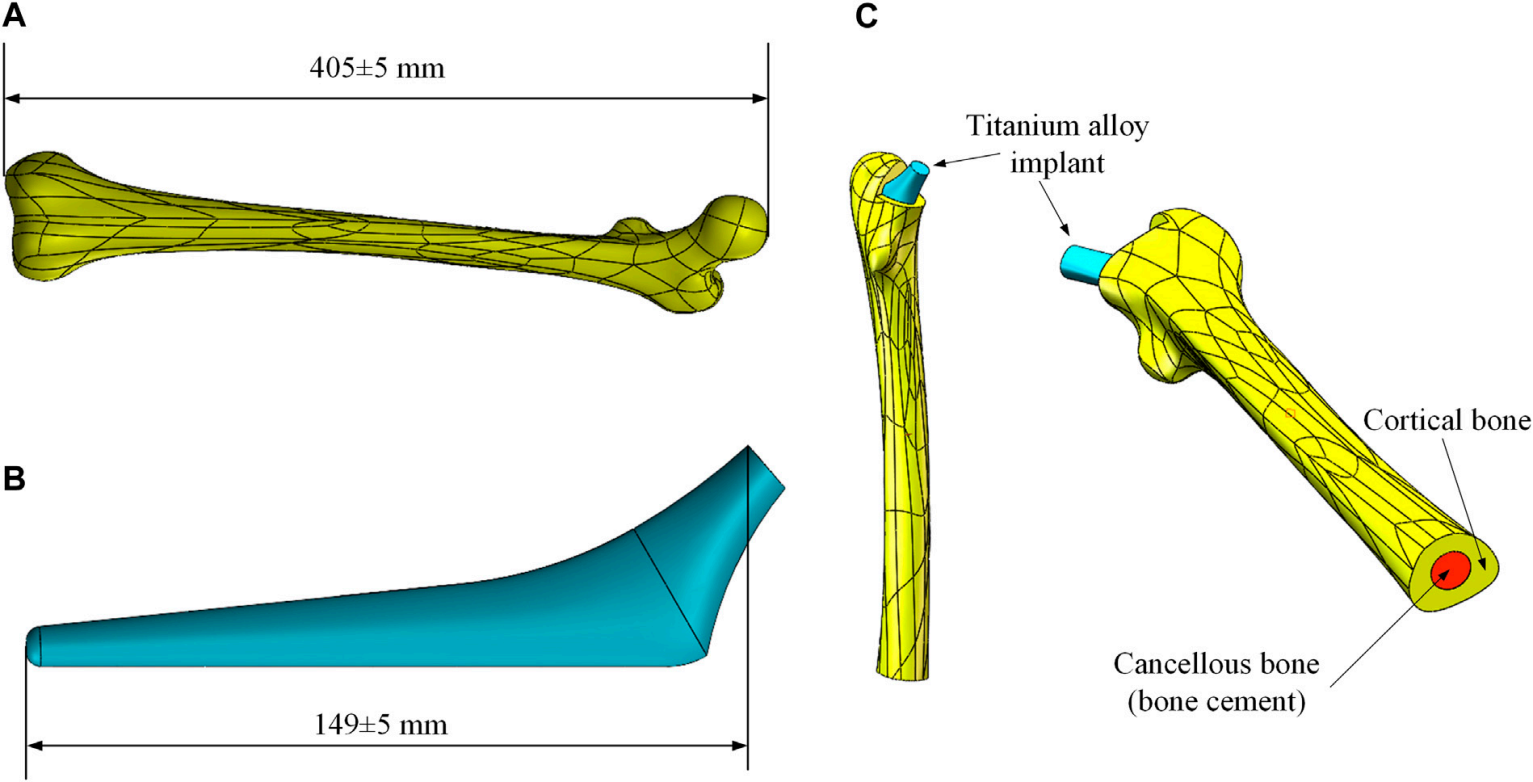
Femur and implant models. **(A)** Femoral model; **(B)** Titanium alloy implant model; **(C)** Combined models.

In clinical practice, the most widely used material for artificial substitute bone is titanium alloy. The aim of this research is to reduce the elastic modulus of the titanium alloy implant by properly designing its gradient porous structure, so that it can adjust the stress distribution of the femur. This way, the stress-shielding effect caused by the modulus inequality will be mitigated. Therefore, it is necessary to develop a three-dimensional solid model for the subsequent parametric design, which comprises the femoral body and the titanium alloy implant. The models used in this study were taken from the GRABCAD (Popular models, 2020), and the selected femoral model and internal implant model were displayed in Figures 1A,B, respectively.

Before the development of the FE model, the excess part of the bone was excised for placing the implant and the cancellous bone. Since the elastic modulus of cancellous bone is similar to that of bone cement, the cancellous bone was replaced with bone cement in this study. Based on the shape of the femur, a solid model of bone cement was designed in the middle of its interior (The red part in Figure 1C). The final femoral model was illustrated in Figure 1C. This model was then imported into FE analysis software for static analysis. The material properties of each part of the model were listed in Table 1.

**TABLE 1:** Material properties of each part (Liu et al., 2019).

Material	Elastic modulus (GPa)	Poisson’s ratio
Ti-6AL-4V	114	0.36
Bone cement (PMMA)	1.6	0.3
Cortical bone	13.7	0.3

**FIGURE 2:**
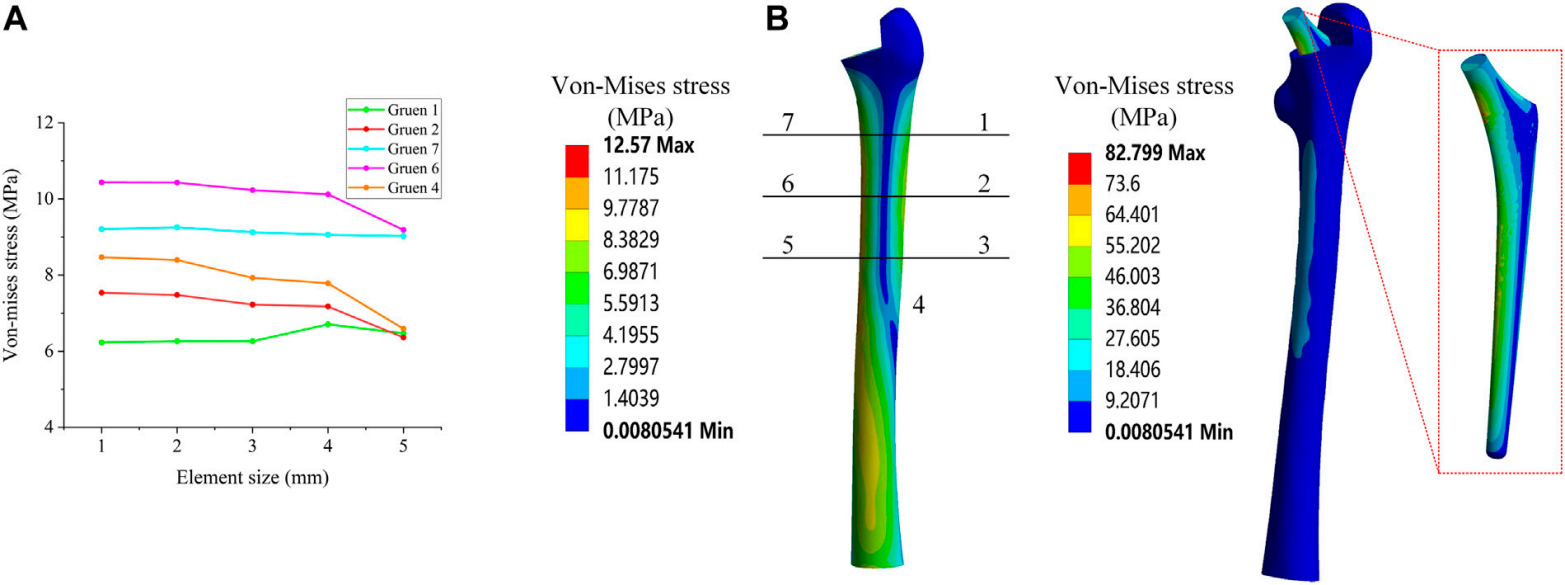
**(A)** Mesh convergence analysis; **(B)** Von Mises stress distribution and Gruen zones partition method.

In this research, a binding contact was adopted. Convergence analysis was performed on the stress under different elements sizes in order to determine which element size was stable and accurate. Five different global element sizes were selected for comparison (Table 2), and the equivalent stress distribution under these sizes when the other conditions were the same was shown in Figure 2A.

**TABLE 2:** Mesh properties for convergence analysis.

Element size (mm)	No. of elements	Average mesh quality
1	230,515	0.75797
2	98,395	0.78819
3	55,036	0.78044
4	34,418	0.75964
5	29,244	0.75425

Figure 2A shows the equivalent stress distribution results, where it could be observed that, when the global element size was greater than 2 mm, the equivalent stress magnitude at each position exhibited a large change. When the global element size was less than 2 mm, the change of the equivalent stress at each position became smaller and more stable. Consequently, in this study, a global element size of 2 mm was used to develop the mesh.

The load of an Asian adult male standing on one leg was considered, as referenced in previous studies (Alkhatib et al., 2019)^,^ (Farmakis et al., 2019). More specifically, a positive pressure of 1200 N was used as the main load acting on the upper end of the implant. As the most commonly used evaluation index in the field of hip implant research, and referring to previous studies (Nam et al., 2019), the equivalent stress of the seven Gruen zones on the surface of the femur were also used as the main evaluation index in this study. The contours of the average equivalent stress distribution calculated by the FE model are presented in Figure 2B.

### FE Model Validation

The content of this section focuses mainly on the experimental validation of the FE model developed in Section 2.1. The materials required for the experiments include: femur, titanium alloy implant, bone cement, strain gauge, and tensile strength test machine. The femur was a composite femur purchased from the Sawbones website (Best Anatomical Medical Training Models Company, 2020) for mechanical experiments, and its mechanical properties were similar to those of human bones. The bone cement was purchased from Heraeus Medical GmbH in Germany, and the titanium alloy implants were made of Ti-6Al-4V through 3D printing.

**FIGURE 3:**
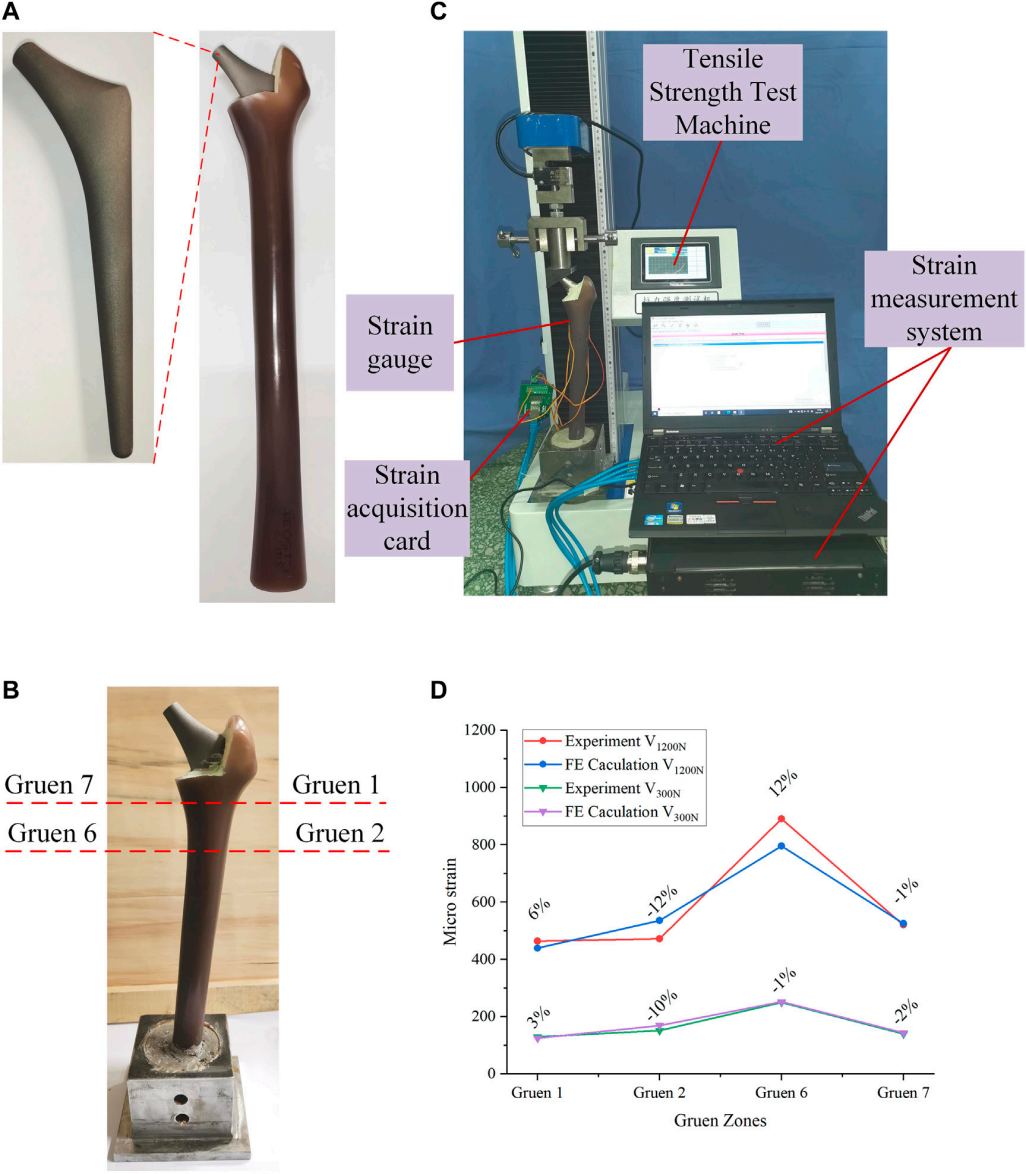
**(A)** Experimental processing of the bionic femur; **(B)** Combination and fixation of the femur and implant; **(C)** The process of validation experiment; **(D)** Comparison of validation experimental results and simulation results.

The processed titanium alloy implant was inserted into the cavity reserved at the upper end of the femur and fixed with bone cement (Figure 3A). After the bone cement solidified, the femur was fixed in the designed base with cement, and was allowed to solidify for 48 h (Figure 3B). In the experiment, a load of 1200 N was applied by the tensile strength test machine, and the strain gauges were used to measure the micro-strain of the four Gruen zones on the outer femur surface. It should be noted that, due to the experimental conditions, the experimental values of all seven Gruen zones could not be measured in this study, so the strain was measured at four Gruen zones and the subsequent optimization process was based on the four Gruen zones used in this experiment. The positions of the four measurement Gruen zones used for simulation and experimentation were calibrated on the basis of a reference point, i.e., a reference point at the same position was selected on the model and the experimental entity, and the subsequent measurement points were calibrated on the basis of this reference point. In addition, another experiment under a load of 300 N was performed in order to compare the experimental results. The experimental loading situation was demonstrated in Figure 3C. The micro-strain of each zone under these two load conditions could be observed in the Supplementary Material. The micro-strain values measured experimentally at the four Gruen zones on the femoral surface were compared with the those calculated by the FE model; thus, the correctness of the FE model was verified based on the magnitude of the error.

The comparison between the experimental and FE analysis results was shown in Figure 3D. Among them, V_1200N_ and V_300N_ represent the micro-strain values when the load was 1200 and 300 N, respectively. As it could be observed in Figure 3D, when the load was 1200 N, the maximum error was 12% and the smallest difference between the micro-strain values was in the Gruen seven zone, where the error was only -1%. In the 300 N load test, the maximum error appeared also in the Gruen 2 zone (10%), while the minimum error was found in the Gruen 6 zone. After experimental measurement and analysis, it was found that the micro-strain value in Gruen 2 was far from the FE result. This may have been caused by a number of reasons, including the lack of a reference coordinate system in the experiment, i.e., the experimental and simulation points may have not been corresponding exactly. Moreover, the relative difference between the numerical and experimental values may have also been caused by the difference between the numerical simulation and the actual boundary conditions at the femur and implant interface (Jetté et al., 2018). At the same time, the use of bone cement may have also affected the results. But in general, the error between the micro-strain values calculated by the FE model and the experimental results was small; thus, the FE model developed in this study could be considered correct and valid.

## Implant Structure Design and Optimization

The FE model was experimentally verified in Section 2, and the parametric design of the porous structure of the implant was based on this model. In this section, the parametric design of the titanium alloy implant was mainly divided into two parts: the determination of the dimension parameter set ( $P_{\text{s}}$ ) of the porous structure based on the Kriging model and the optimization of the gradient elastic modulus based on the response surface methodology.

**FIGURE 4:**
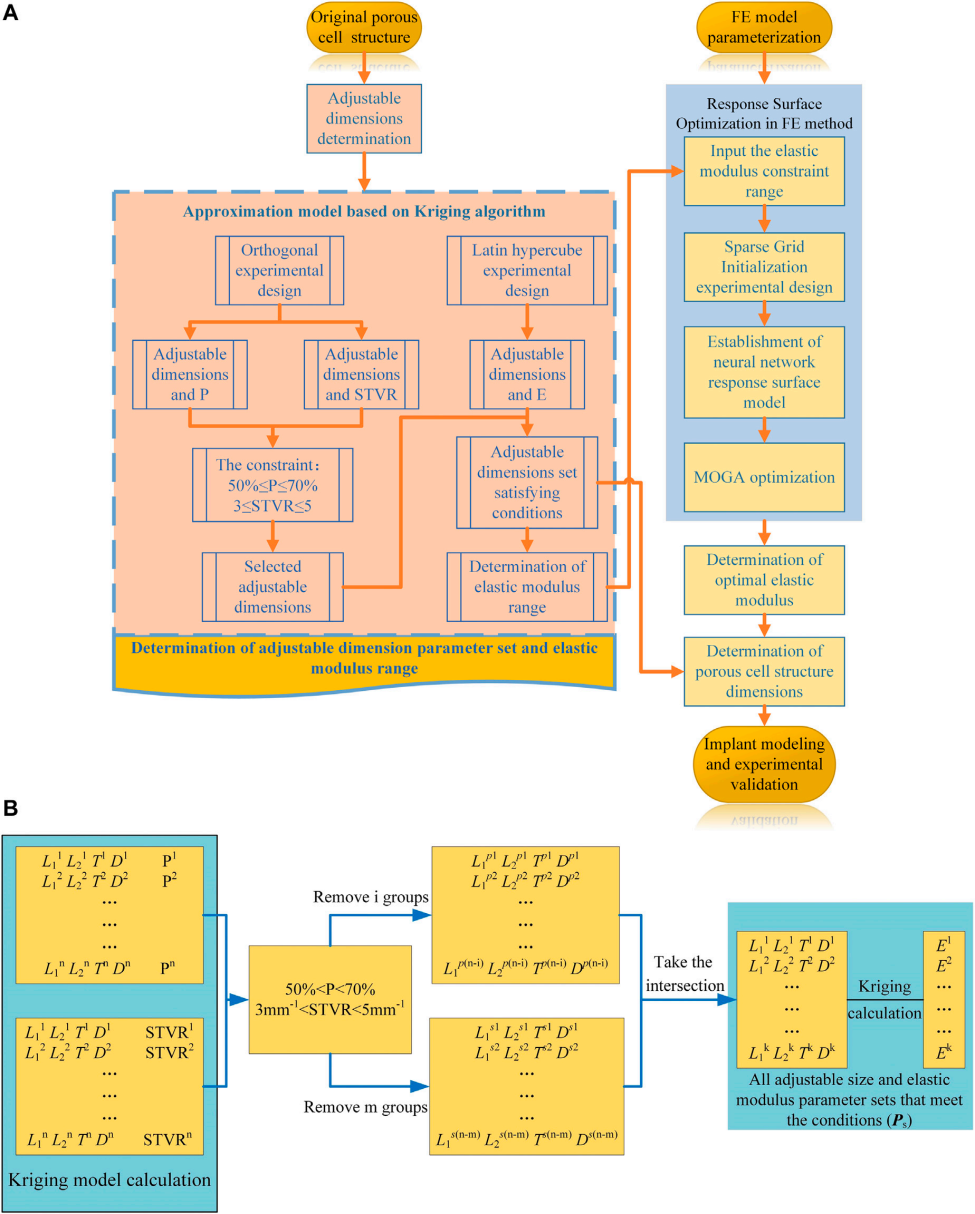
Optimization process. **(A)** Flowchart of the implant design and optimization process; **(B)** Process of parametric optimization based on Kriging model.

In the process of parametric optimization in this section, the mapping relationship between the internal dimensions of the cellular structure and the equivalent stress on the femoral surface cannot be directly established by the FE method. Moreover, compared with the FE method, the Kriging method can also perform personalized optimization according to the constraints of the cell. Therefore, in order to improve the efficiency of optimization and realize the parametric design of porowus structures, the respective advantages of the two methods were combined for the design of implants with gradient moduli in this study. In order to express the mapping relationship between various parameters, the Kriging approximation model was developed based on the original porous cell structure, and the dimension parameter set for subsequent optimization design was determined. Specifically, the Kriging model was developed to express the relationship between adjustable dimensions and STVR, porosity and elastic modulus, and the adjustable dimensions set and the set of elastic modulus for subsequent optimization were derived from these relationships. The upper and lower limits of the adjustable dimension parameter set satisfying the conditions (Eqs 3 and 4) was used to determine the upper and lower limits of the elastic modulus to be input into the response surface optimization range settings, and then the elastic modulus was optimized by the response surface optimization provided by the FE method (Figure 4A). The optimal equivalent elastic modulus of each segment could be determined when the conditions of Eq. 2 were satisfied. The dimension parameters of each cell were determined in the adjustable dimension parameter set ( $P_{\text{s}}$ ) based on this optimal elastic modulus and finally the optimal dimensions were modelled and experimentally tested. The specific implementation process was illustrated in Figure 4A. The formulation used for optimization, which is based on linear static FE analysis, and the specific parametric optimization process based on this optimization formula was shown in Figure 4B.

The general optimization algorithm is defined as follows:

$$\text{Find}\;E=\left[E_{1},E_{2},E_{3},E_{4},E_{5},E_{6},E_{7}\right]$$ (1)

$$\text{Max}\;\sigma =\left[\sigma_{1},\sigma_{2},\sigma_{3},\sigma_{4}\right]$$ (2)

The constraints are as follows:

I Porosity

50%≤P≤70% (3)

II Surface-to-volume ratio

$$3\text{m}{\text{m}}^{-1}\leq STVR\leq 5\text{m}{\text{m}}^{-1}$$ (4)

III Equivalent elastic modulus

$$E_{i}\in \left\{P_{s}\right\}$$ (5)

where ***E***, ***σ***, and $P_{\text{s}}$ are the equivalent elastic modulus of each implant segment (Implant was divided into eight segments, which will be explained in detail later), the equivalent stress on the outer surface of the femur (The zone where the equivalent stress is located was consistent with the zone used for the experimental measurement), and an adjustable dimension parameter set, respectively. The stress-shielding effect is mainly due to the high elastic modulus materials that bear most of the stress, resulting in the stress acting on the femur less than required for normal growth. Therefore, the stress-shielding effect can be mitigated by increasing the stress stimulation on the femur. However, due to experimental conditions, increasing the stress on the outer surface of the femur will be the goal of optimization (Eq. 2).

### Determination of the Dimension Parameter Set Based on the Kriging Model

**FIGURE 5:**
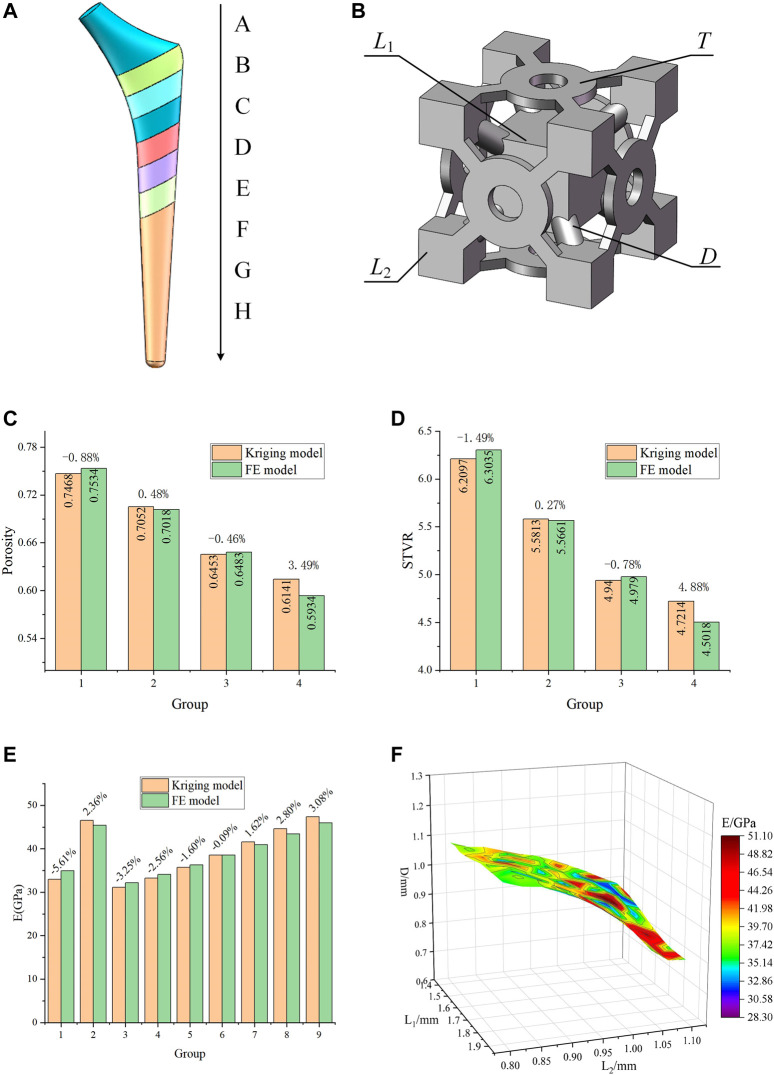
Designed porous structure and error analysis of the Kriging model. **(A)** Segmented implant; **(B)** Initial cell structure and adjustable dimensions; **(C)** Error of porosity calculated by the Kriging model and the FE model; **(D)** Error of STVR calculated by the Kriging model and the FE model; **(E)** Error of elastic modulus calculated by the Kriging model and the FE model; **(F)** Relationship between *E* and the three groups of dimensions in the dimension parameter set.

In order to satisfy the optimization goals for the different femur positions, the solid model of the titanium alloy implant was divided into eight segments from A to H (Figure 5A). In the A segment, which was the main force-bearing part, titanium alloy entities were used. The elastic modulus of the other seven segments varied according to the optimization results. In order to simplify the optimization process and guarantee the adjustability of the structure, a BCC cell able to individually change the local dimensions was designed (Figure 5B). This type of structure is universal, and the subsequent optimization was based on this structure. By changing its internal dimensions, each segment could obtain a suitable elastic modulus.

Considering the requirements for bone in-growth and manufacturability, Chen et al. (2020) reported that a scaffold with a porosity of 60% has the best cell proliferation and osteogenic differentiation (*in vitro* experiment) and bone in-growth (*in vivo* experiment). Jetté et al. (2018) suggested that, when designing porous structures, the porosity should be between 40 and 70%. Abate et al. (2021) showed that the cellular implants with porosity of 56 and 58% have the potential to be used in orthopedic and prosthetic applications to improve osseointegration. When using BCC structures, the porosity in the range of 50–70% can be used to design gradient porous implants so that the mechanical properties of cortical bone can be simulated (Chowdhury et al., 2021). At the same time, bone growth, migration, and cell adhesion are also affected by the surface-to-volume ratio (STVR) of the porous structure. Studies by Beaupré (Beaupre et al., 1990) and Coelho (Coelho et al., 2009) had indicated that a tight bone fixation could be provided by the implant if the STVR of their porous structure was in a range of 3–5 mm^−1^. Therefore, in this study, a porosity ranging within 50–70% and an STVR ranging within 3-5 mm^−1^ were used as constraints for the optimization of the porous cell structure dimensions. The porosity and STVR should satisfy the above constraints by changing the adjustable dimensions of the porous structure. According to the original structure in Figure 5B, the freely and independent adjustable dimensions were the side length *L*_1_ of the center cube, the side length *L*_2_ of the outer cube, the pillar diameter *D*, and the thickness *T* of the connecting plate. Therefore, only the mapping relationships between the four dimensions and P, STVR were needed in order to determine all the structural dimensions that satisfy the above constraints.

In order to determine these mapping relationship, two Kriging approximation model were developed, where the four adjustment dimensions were used as the input parameters, P and STVR were used as the output response parameters, and the genetic algorithm was used to optimize the Kriging model parameters and increase its precision. In order to develop a Kriging model, it is necessary to design orthogonal experiments for the input and output parameters. This method can make the role of each factor clear, and can pick out representative test points for experiments to find the best level matching, and can greatly reduce the number of experiments, and there is no strict limit on the number of factors. The optimization process of the Kriging model and the results of the orthogonal experiment were provided in the Supplementary Material. In this study, a total of 16 sets of experiments with four factors and four levels were used and the model was determined based on the results of the orthogonal experiments. Four groups of adjustment dimensions that were not trained by the Kriging model and completely different from those listed in the Supplementary Material were selected to compare the actual value calculated by the FE model with that predicted by this model and evaluate the accuracy of the model. The results were presented in Figures 5C,D.

As it can be observed in Figures 5C,D, the error between the results predicted by the developed Kriging model and those obtained by the FE simulation was small. Regardless of whether it was the predicted porosity or STVR, after inputting the same dimensions parameters, the maximum error between the results obtained by the Kriging model and the FE model appeared in Group 4. The reason is that when one or more of the four adjustable dimensions are too large or too small, the design deviation will increase significantly. Nevertheless, the maximum error within the dimension design range was only 4.88%, and this model could be considered accurate. All manufacturable adjustable dimensions were input into the model, and the porosity and STVR values corresponding to all manufacturable adjustable dimensions could be determined through the Kriging model. After removing the values that do not satisfy the constraints (Figure 4B), the adjustable dimensions set that satisfy the conditions could be obtained.

Similarly, the mapping relationship between the adjustable dimensions and the elastic modulus of this structure also needs to be expressed through a Kriging model. Different from the previous experimental design method, since the relationship between adjustable dimensions and elastic modulus is more complicated, the design of experiment needs to be completed with the help of FE method. After its parameterized modeling, the original porous structure was imported into the ANSYS, which was used to calculate the deformation values. The adjustment dimensions were used as the input parameters and the deformation values were used as the output parameters. Subsequently, the Latin hypercube experimental design method was used to obtain 25 groups of experiments (Supplementary Material). In order to establish the mapping relationship between the adjustment dimensions and the elastic modulus, it is necessary to convert the deformation values into the elastic modulus, as described by Eq. 6. The Kriging model between the adjustable dimensions and the elastic modulus was determined through these 25 groups of experimental data.

E=FSΔLL (6)

where *E* is the elastic modulus of the porous structure, *F* is the force acting on the porous structure, *S* is its cross-sectional area, ΔL is the amount of deformation, and *L* is its total length in the direction of deformation.

In order to verify the accuracy of this model, nine groups of untrained adjustable dimensions were selected, and the actual value calculated by the FE model was compared with that predicted by the model. The error of this model was evaluated as shown in Figure 5E, where it can be observed that the maximum error between the elastic modulus predicted by the Kriging model and that calculated by the FE model was only 5.61%; thus, this model could be considered as accurate. The previously obtained values of all the eligible adjustable dimensions were substituted into this Kriging model to calculate the elastic modulus. Therefore, the elastic modulus range used for subsequent optimization and its corresponding adjustable parameter set ( $P_{\text{s}}$ ) were determined by the constraint conditions of P and STVR. Among them, all the dimension parameter sets were discrete. Three sets of ***L***_1_, ***L***_2_, and ***D*** among the four sets of dimension parameters and the calculated elastic modulus ***E*** were selected to express this dimension parameter set ( $P_{\text{s}}$ ), as shown in Figure 5F.

### Elastic Modulus Optimization Based on the Response Surface Method

In Section 3.1, the elastic modulus range that satisfies the constraints was determined through the Kriging model. In this section, the response surface optimization of FE method was used to select from that range appropriate elastic modulus values for the implant.

After the FE model had been parameterized, the elastic modulus was set as the input value, and the equivalent stress as the output value. The response surface optimization was used to perform the final optimization design. Response surface optimization comprises mainly three parts: experimental design, approximation model development, and genetic algorithm optimization. In this study, the extremes of the elastic modulus range obtained in Section 3.1 were set as the upper and lower limits of the input values that need to be defined during the experimental design of the parameterized model. The sparse grid initialization method was used to determine the experimental samples in the experimental design, one advantage of sparse grid initialization is that it refines only in the directions necessary, so that fewer design points are needed for the same quality response surface and another is that it is effective at handling discontinuities. A neural network approximation model was developed using these samples in the response surface type selection. Multilayer perceptron neural networks is used in the neural network model inside workbench, this model works well for highly nonlinear responses and is suitable for use when the number of input parameters is high. Finally, the multi-objective genetic algorithm (MOGA) was used to optimize the elastic modulus based on the approximation model, and the maximum value of the equivalent stress on the femoral surface was taken as the target for optimization. Through the above optimization design method, the elastic modulus of the reference point used for the design of the implant and the corresponding equivalent stress value of the femoral surface were obtained.

**FIGURE 6:**
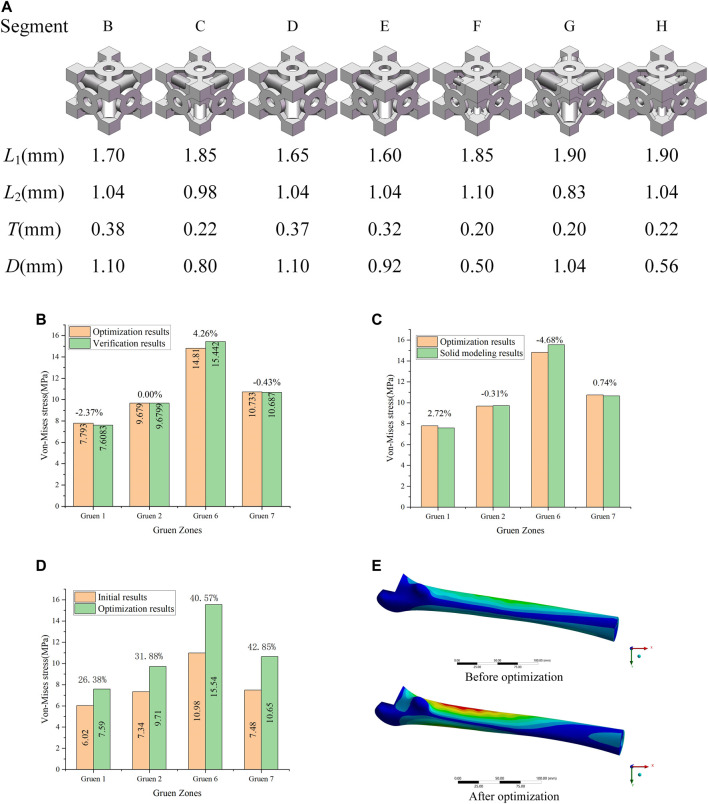
**(A)** Porous structure of the B-H segment; **(B)** Validation of optimization results; **(C)** Error between the optimization of the elastic modulus and the solid modeling after optimization; **(D)** Optimization effect analysis; **(E)** Equivalent stress distribution of the femur before and after optimization.

As it can be seen in Table 3, the elastic modulus of each segment of the optimized implant was different, and the elastic modulus of Segments B and D were larger. In order to verify the effectiveness of this optimization method, the elastic modulus values listed in Table 3 were added to the FE model to perform simulations. The simulation results were compared with the equivalent stress in Table 3, and the comparison results were shown in Figure 6B. It could be observed that the difference between the equivalent stress obtained after optimization and that calculated by the actual elastic modulus was not large, and the maximum error was 4.26%, which was in line with the expectations.

**TABLE 3:** Optimization results.

Implant segment	Elastic modulus (GPa)	Gruen zones	Von mises stress (MPa)
B	50.669	Gruen 1	7.793
C	29.814	Gruen 2	9.679
D	51.047	Gruen 6	14.810
E	39.613	Gruen 7	10.733
F	28.465	—	—
G	31.316	—	—
H	28.717	—	—

The dimensions of each part corresponding to the optimized elastic modulus in Table 3 could be found from the adjustable dimension parameter set ( $P_{\text{s}}$ ) in Section 3.1 and the solid model of the porous structure determined by these dimensions was designed. The porous structure of the B-H segments could be obtained according to the optimized adjustable dimensions, as shown in Figure 6A. Based on the porous structure model, the actual elastic modulus of the structure designed according to the optimization results can be calculated, and then, the corresponding equivalent stress on the femoral surface could be obtained from the FE model. The prediction results generated by the Kriging approximation model will produce errors, and errors may also occur during modeling. Consequently, it is necessary to consider the cumulative error of the equivalent stress predicted by the Kriging model; that is, the error between the optimized equivalent stress and the equivalent stress of solid modeling based on the Kriging model predictions. This error was presented in Figure 6C. At the same time, in order to comprehend the actual effect of the optimization method involved in this research, the equivalent stress obtained by this method was compared with that of the initial femoral surface without optimization, and the results were shown in Figure 6D.

It can be observed that the equivalent stress on the femoral surface calculated by the FE model was not much different from the optimized result, and the maximum error was within 5%. Consequently, the method proposed in this study to optimize the porous structure of the implant was effective, and the error produced was small. According to the comparison between optimized and initial results (Figure 6D), the equivalent stress on the femoral surface was increased by at least 26% after optimization compared to the unoptimized results. The best optimization effect was in Gruen 7, where the equivalent stress was increased by 42.8%. In addition, it could be seen from Figure 6E that compared with the equivalent stress of the femur before optimization, the equivalent stress of the femur after optimization increases significantly, and the area where the stress increases was also larger.

Through the mutual validation of the FE model, the implant with porous structure optimized by the proposed method also exhibited good results in the calculation, and the equivalent stress on the surface of the femur was improved.

## Experimental Results

**FIGURE 7:**
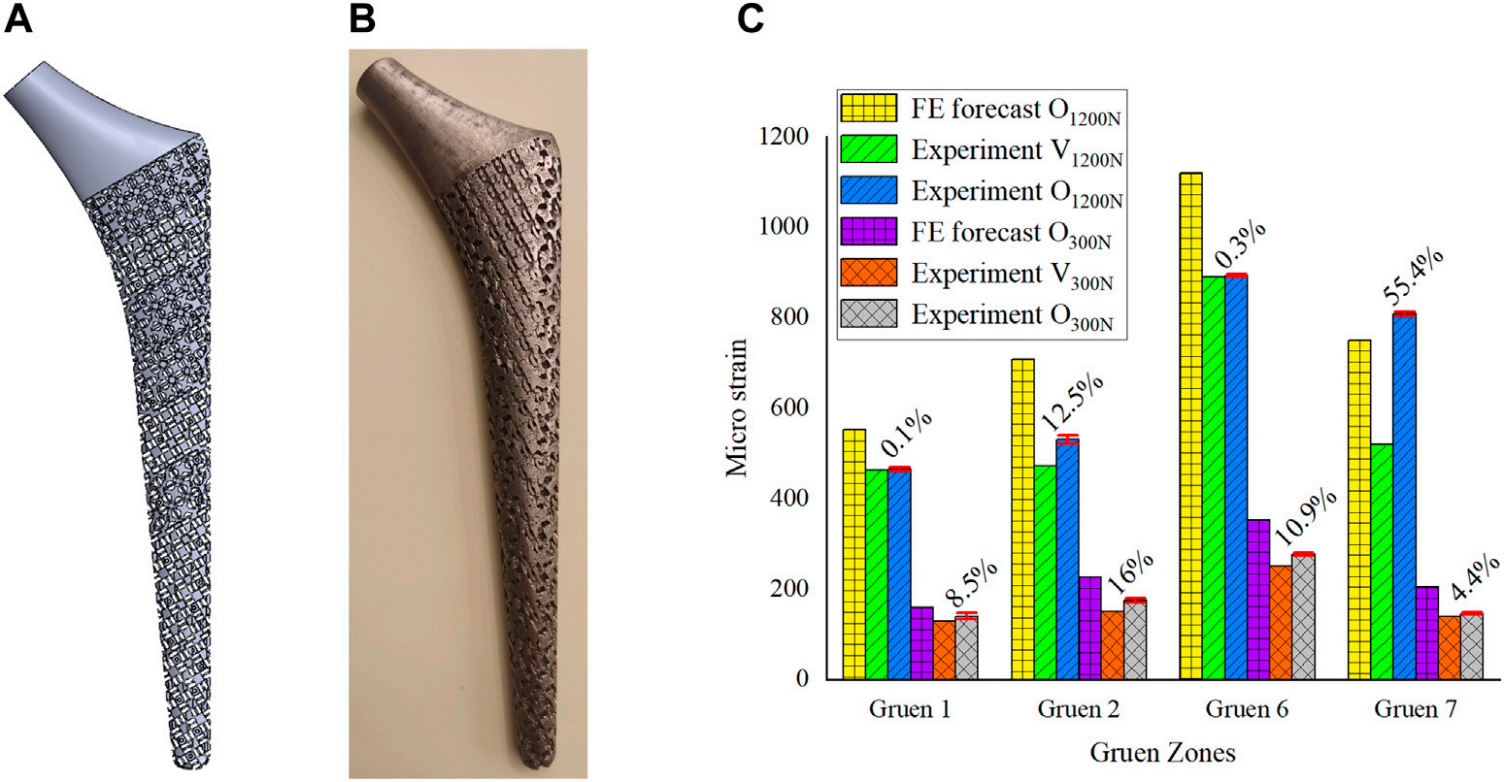
**(A)** 3D model of gradient porous implant; **(B)** The hip implant manufactured by 3D printing; **(C)** Analysis of the optimization effect.

In Section 3, the porous cell structures were parametrically designed and the Kriging model and FE method were used to obtain a set of dimensions parameters that satisfy the constraints (Figure 6A). After optimizing the elastic modulus, the optimal porous structure model of each segment of the titanium alloy implant was developed (Figure 7A). In order to verify the actual effect of this optimization method, the experimental analysis was performed again with full reference to the model validation experiment performed in Section 2.2. In order to ensure the validity of this optimization effect analysis experiment, 10 measurements were taken for this optimization experiment and performed statistical analysis on these 10 experiments as shown in Figure 7C. The 3D printed implant used in the experiments was presented in Figure 7B and the experimental data were provided in the Supplementary Material. The effect of the optimization results obtained by the experiment was depicted in Figure 7C.

In Figure 7C, V_1200N_ is the validation experimental result before optimization (Section 2.2), O_1200N_ is the experimental result after optimization, and there are also optimization results predicted by the FE model. The same is true for the experiment under a load of 300 N. From the statistical analysis obtained from 10 experiments in Figure 7C, it could be seen that the results of multiple experiments performed in this optimization experiment were relatively stable, and it could be observed that the experimental measurement results after optimization were better than those before optimization. The micro-strain on the femoral surface was increased by 17.1% on average under 1200 N load and by 10% on average under 300 N load. The most obvious improvement in both cases was still at Gruen 7, which was the same as the FE prediction. Under the load of 300N, the implant was firmly attached to the femur by the bone cement, and the stress stimulation on the femur was relatively uniform, so it could be seen that the micro strain on the femoral surface increased more evenly. However, at a load of 1200 N, due to the high load applied to the implant, the force state between the implant and the femur is similar to that of a lever, i.e. Gruen 2, the support point, and Gruen 7, the stress point, are the main load-bearing areas, while Zones 1 and 6 are unstressed or less stressed, which results in a non-uniform growth of micro strain on the femoral surface as shown in Figure 7. In addition, due to some of the reasons mentioned in Section 2.2 and the manufacturing errors of 3D printed porous implants, the experimental results were generally inferior to the FE simulation ones.

## Conclusion

In this paper, a new parametric design method was proposed to mitigate the stress-shielding effect by optimizing the gradient porous structure of implants. A FE model of a hip implant for elastic modulus optimization was developed and validated experimentally. The porous structure was parametrically designed, the mapping relationship between the adjustable dimensions and porosity, STVR, and elastic modulus was established, and the adjustable dimensions set and the set of elastic modulus for subsequent optimization were derived from these relationships. These parameter sets calculated by the Kriging model were used as the constraints for the subsequent optimization of input and output parameters. By combining the results calculated by the kriging model, an optimal implant with porous structure and gradient elastic modulus was designed by the FE method. The experimental results demonstrated that the femoral surface micro-strain was increased by 17.1% and 10% on average compared with the unoptimized results. Consequently, it can be concluded that the parametric optimization design method proposed in this paper was effective and could substantially mitigate the stress-shielding effect by reducing the elastic modulus of the implant. The parametric design method proposed in this research is based on a general structure, the local dimensions of which can change individually; thus, this method is suitable for various situations where the elastic modulus needs to be adjusted through porous structure parametric design. In the future, the method of parametric design in this paper will be used to study the differentiation of the porous structure of implants in femurs of different ages.

## Data Availability

The original contributions presented in the study are included in the article/Supplementary Material, further inquiries can be directed to the corresponding authors.
