# Supplementary material for: Parametric Design of Hip Implant With Gradient Porous Structure
Source: Front Bioeng Biotechnol. 2022 May 16;10:850184. doi: 10.3389/fbioe.2022.850184 (PMC9150022; doi:10.3389/fbioe.2022.850184)
Supplement: Supplementary file 1 [file DataSheet1.doc]

**Supporting Information**

**Experiment - model validation**

Two experiments were performed by applying two different load levels, and the validity of the FE model was verified by comparing the experimentally-measured micro-strain values with those calculated by the FE model (Fig. S1). In order to ensure the consistency with the FE model and overcome the limitations of the experimental conditions, in each experiment, the Z-direction micro-strain values on the surface of the four Gruen zones were measured under the same conditions.

(a) 300N (b) 1200N

Fig. S1 Micro-strain values measured in the model validation experiment

**Experiment - optimization effect analysis**

The process and all settings of this experiment were consistent with those of the model validation experiment. The only inconsistencies were: the use of optimized porous titanium alloy implants, and some difficult-to-control factors, such as the location of the strain gauges, and the bone cement solidification status. Besides, in order to ensure the validity of the experimental result, we conducted 10 experiments for optimization effect analysis, and performed statistical analysis on these 10 experiments. The measurement results of these 10 experiments are shown in Fig. S2 respectively.


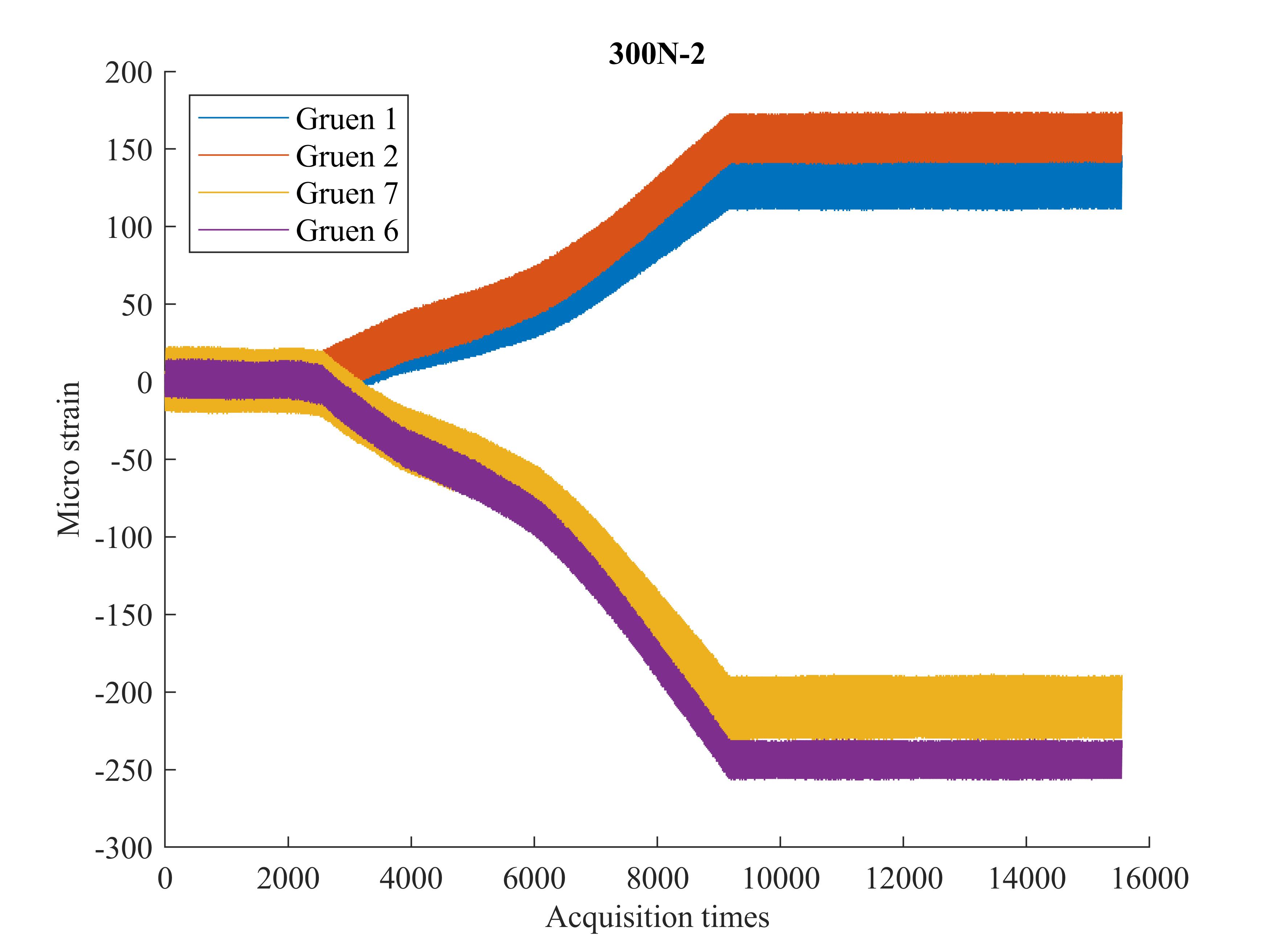

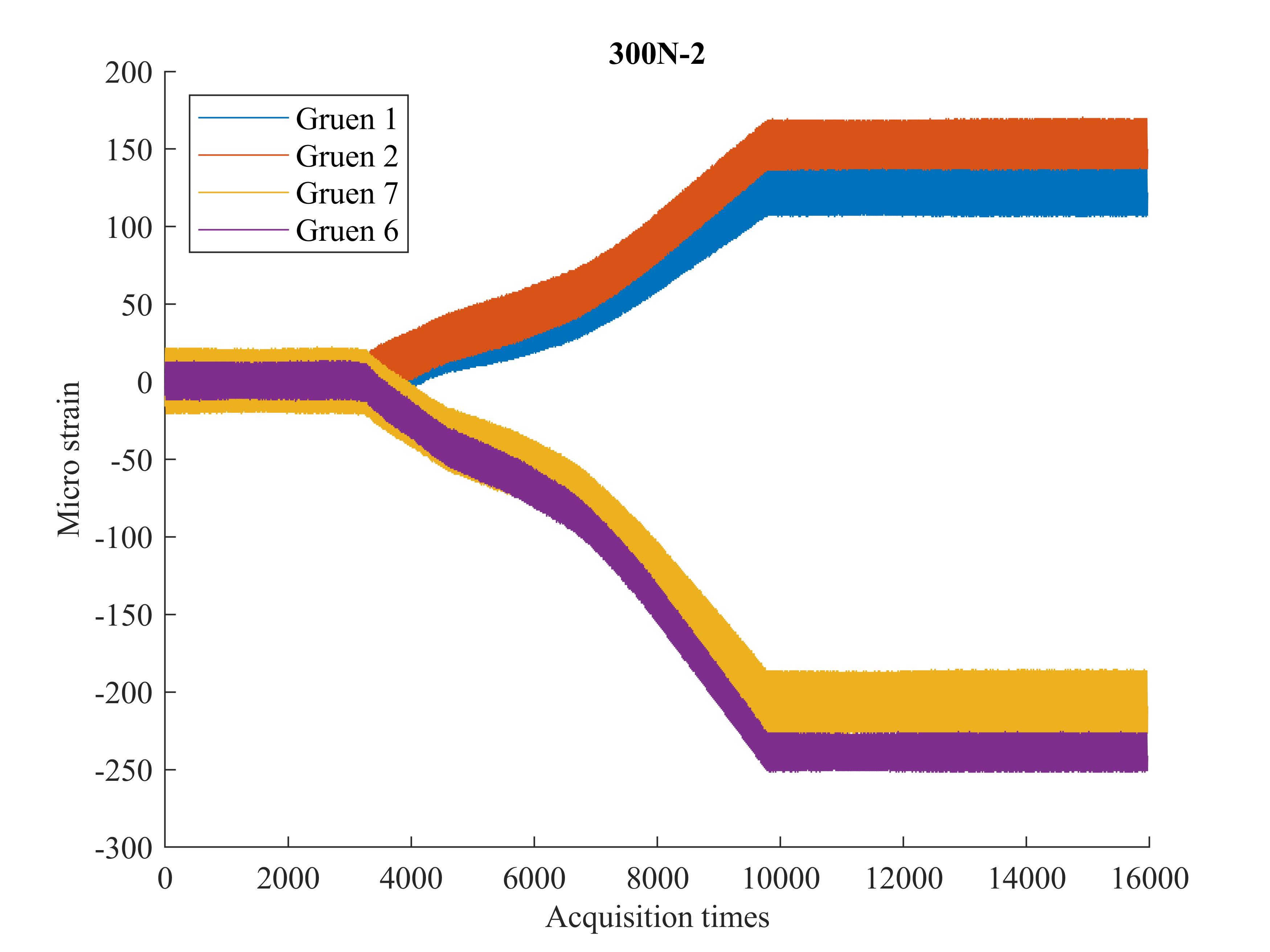

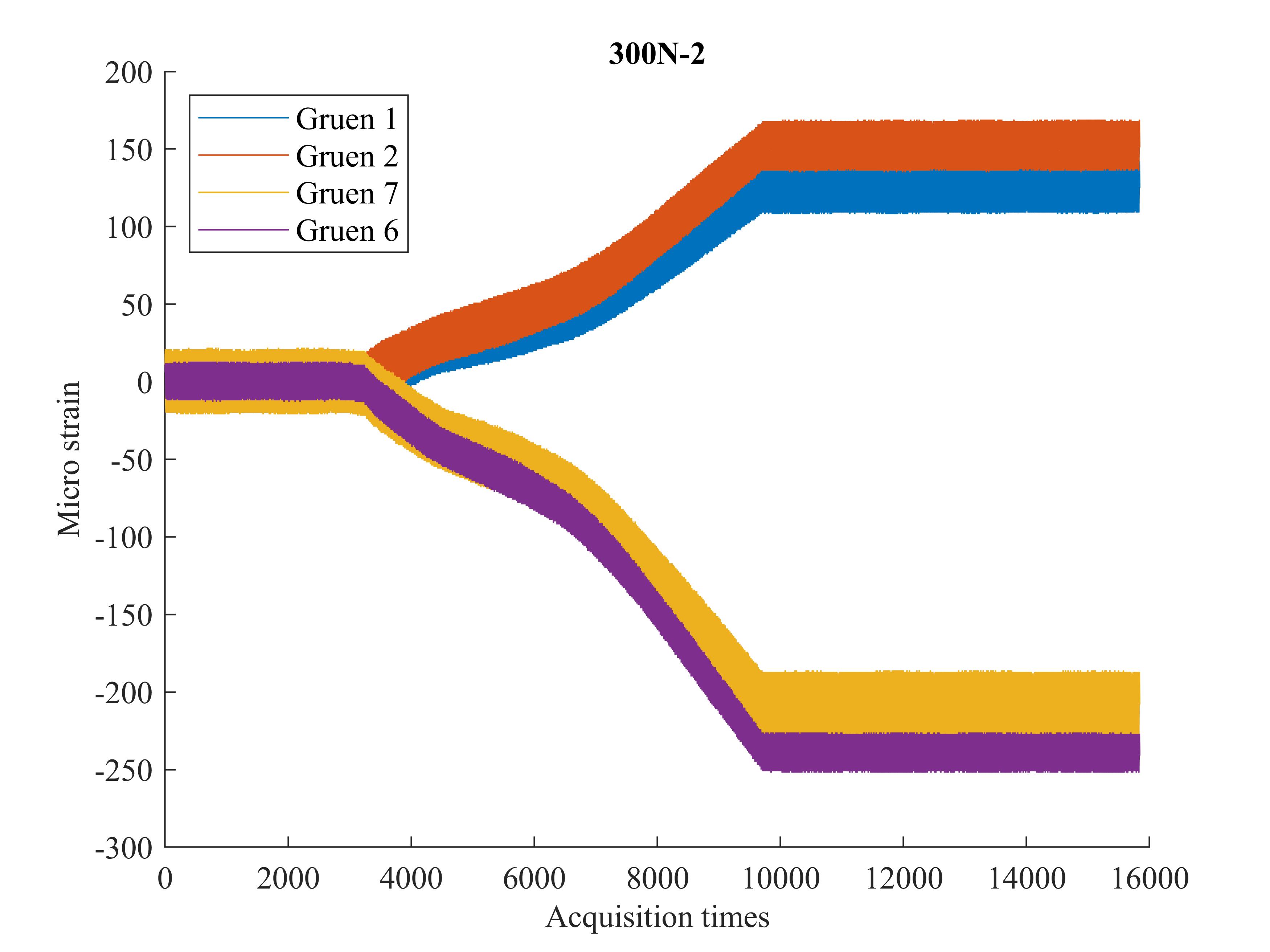

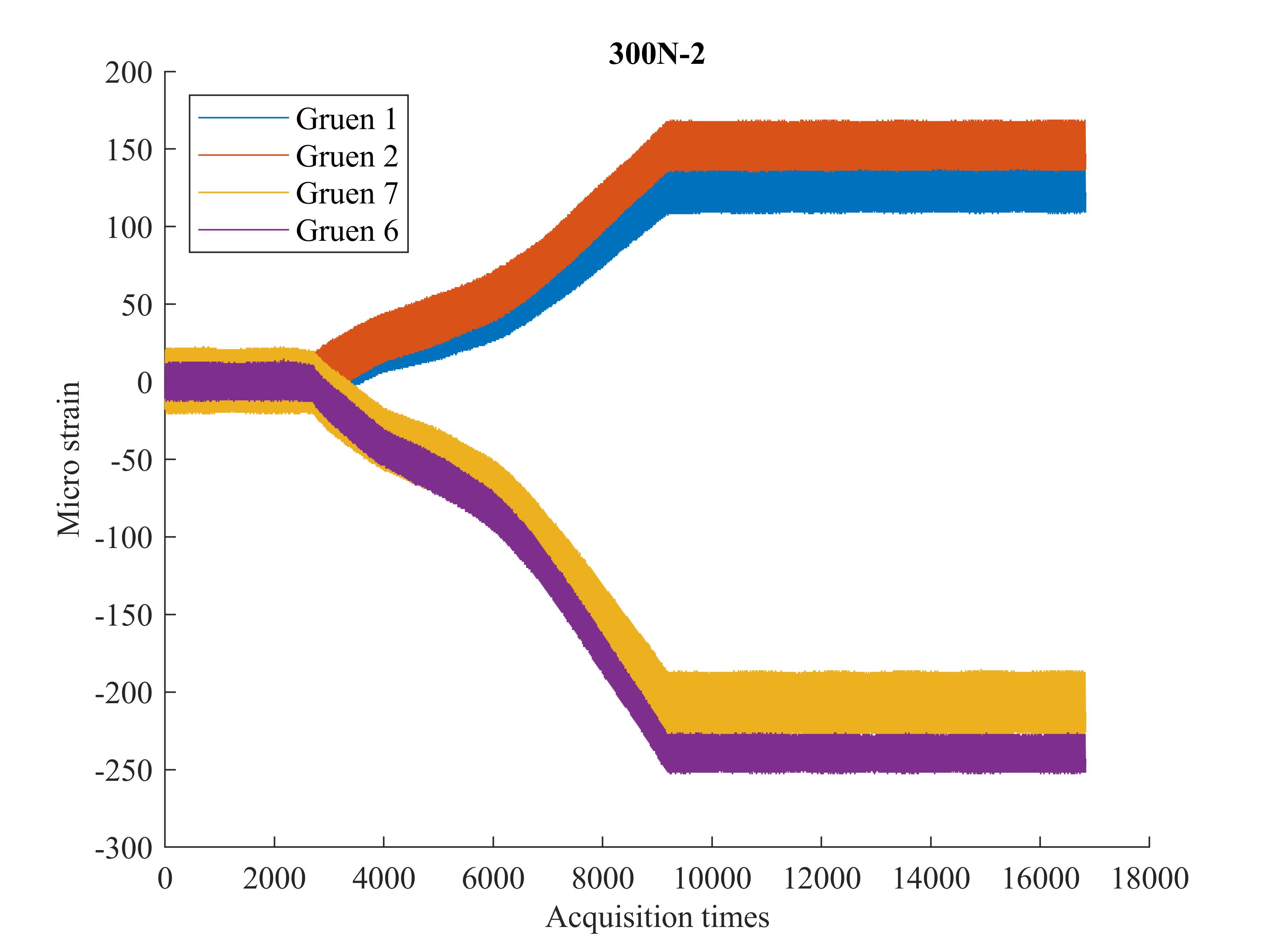

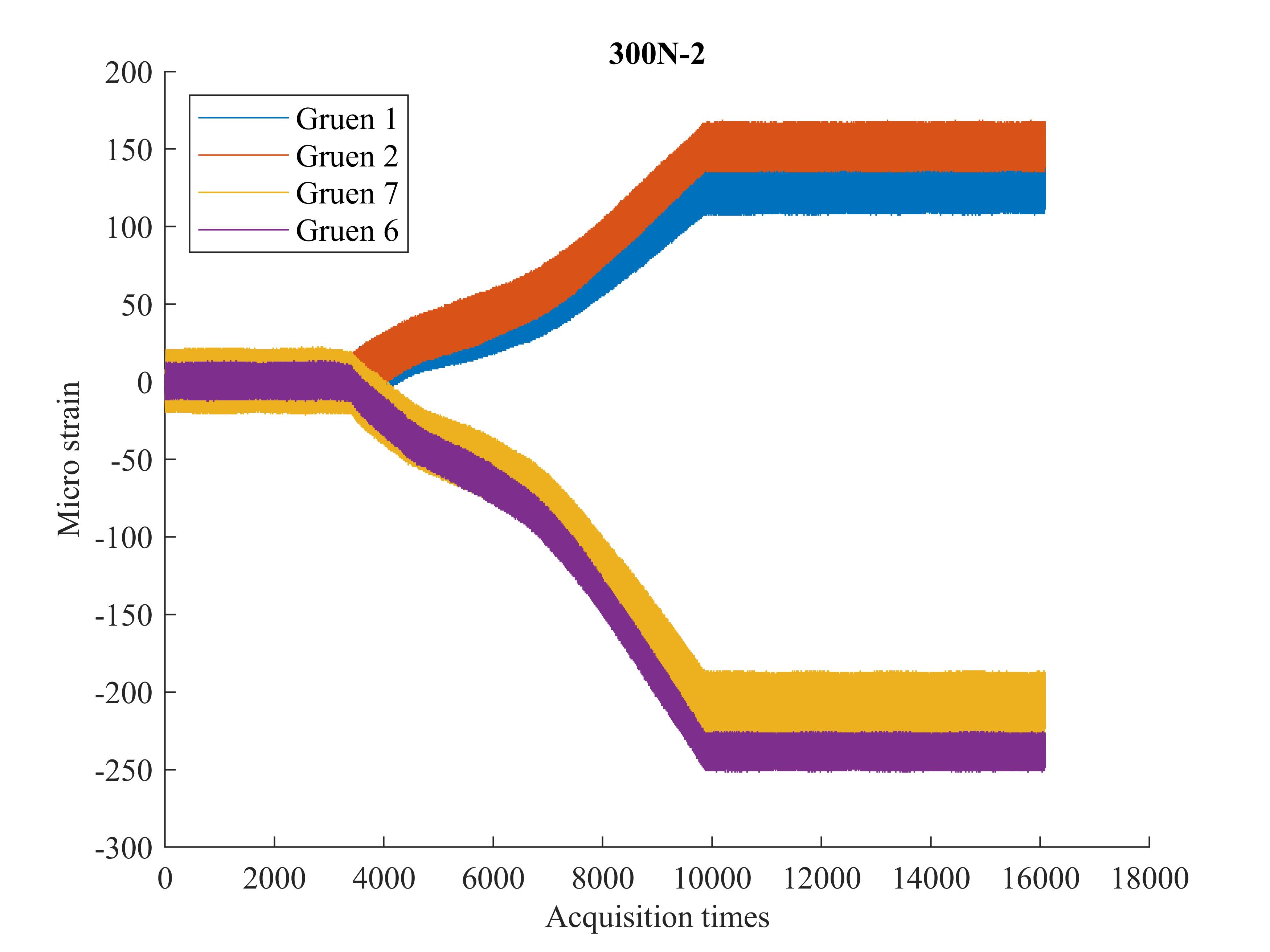

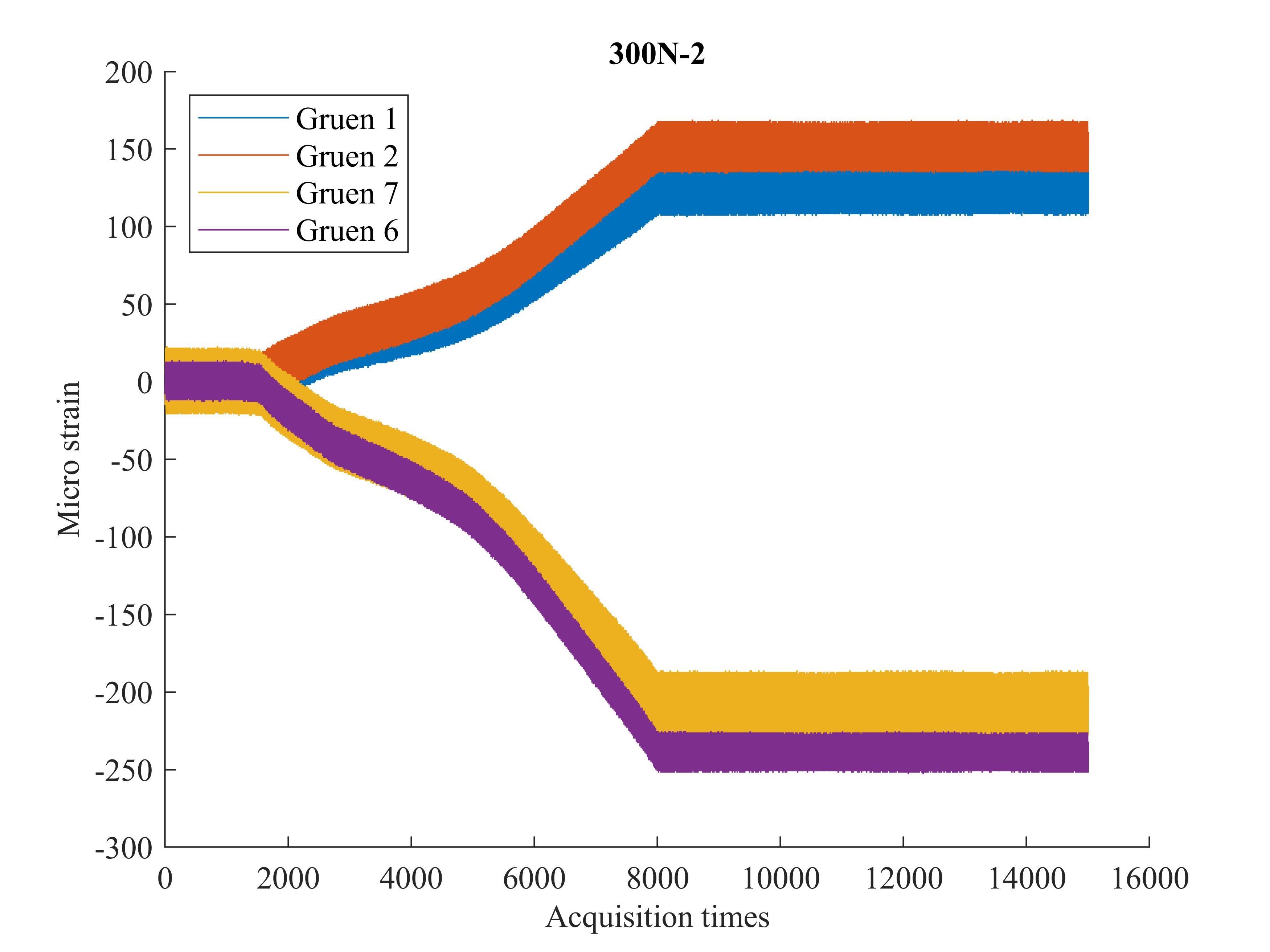

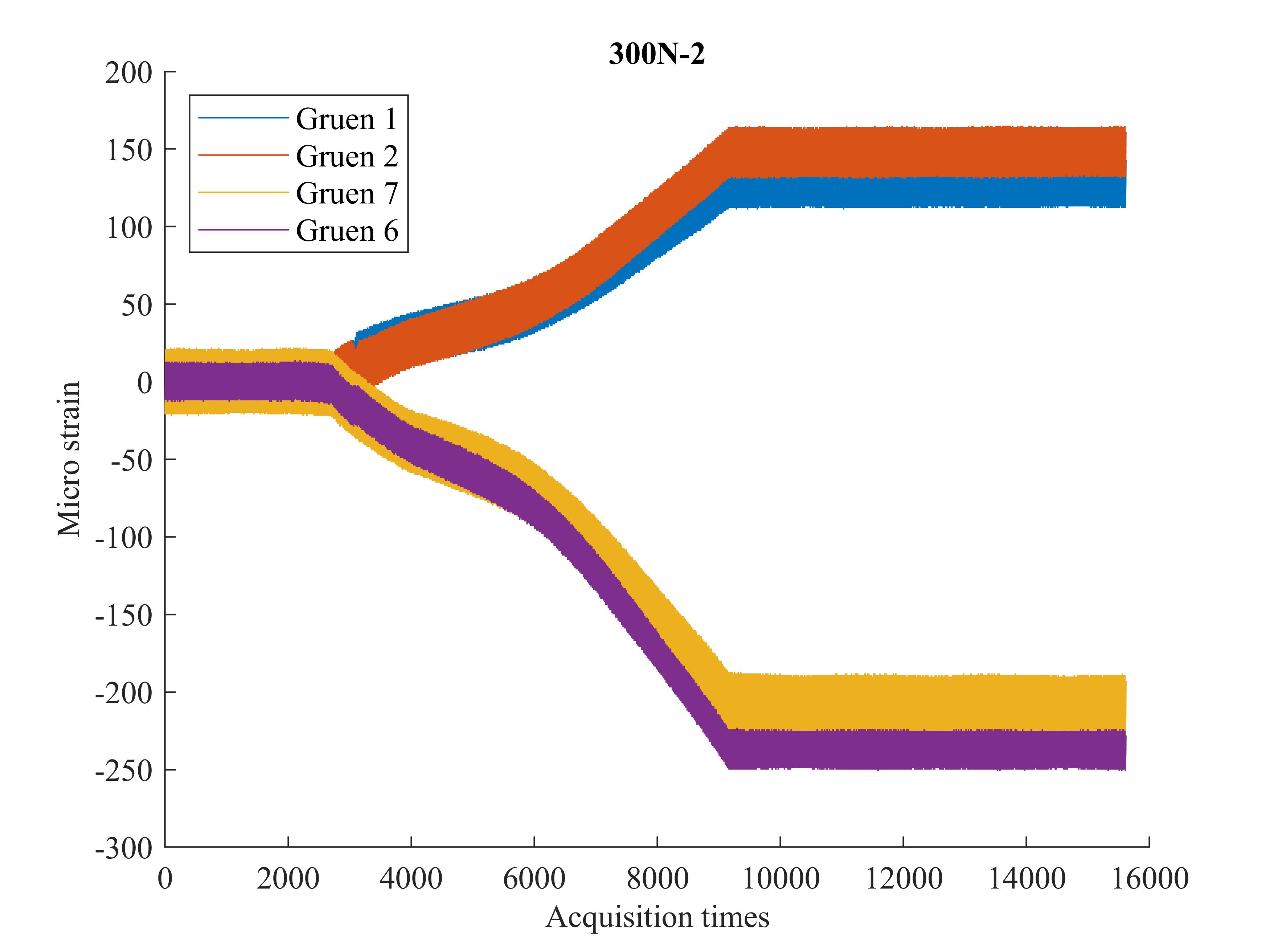

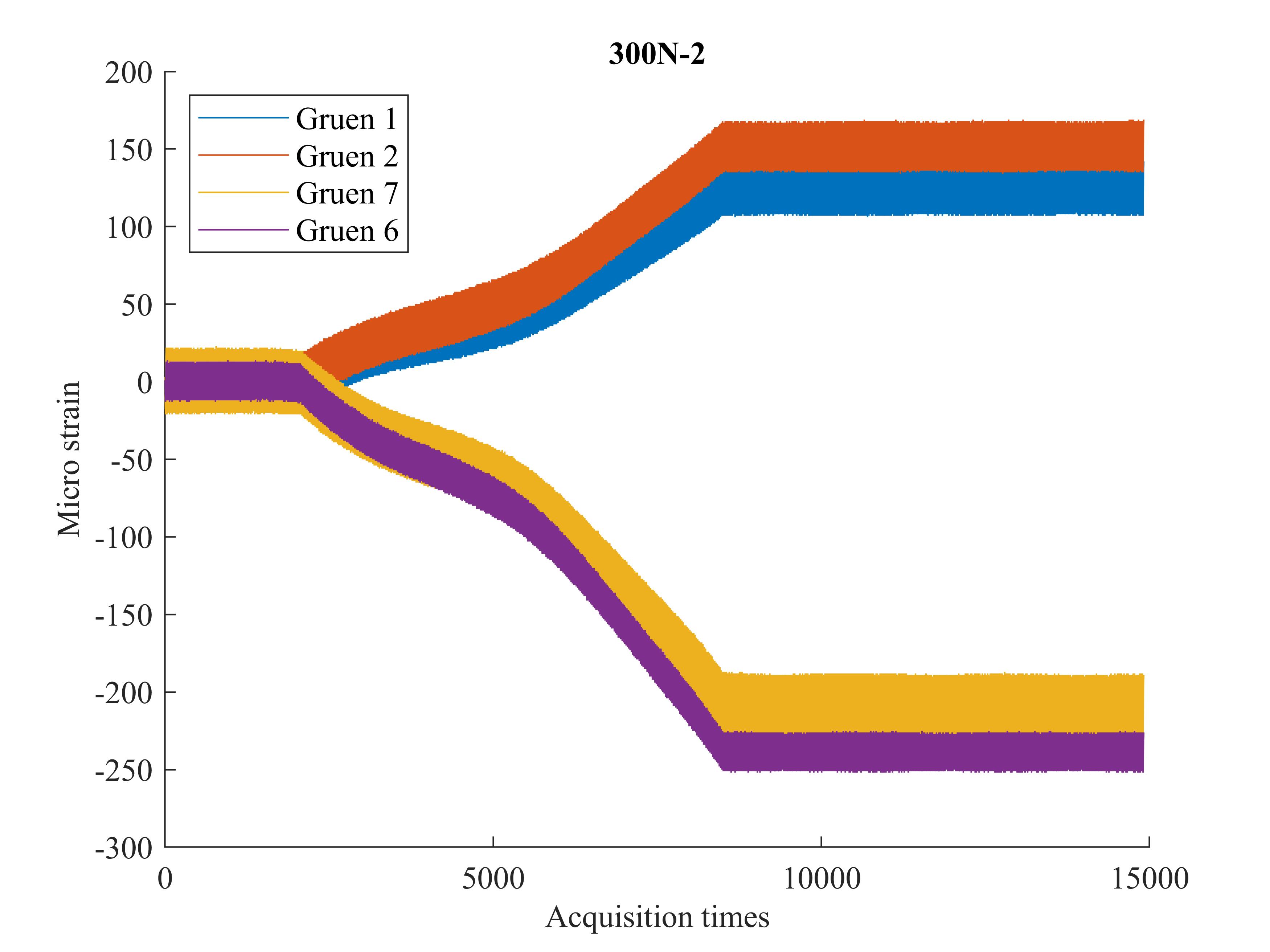

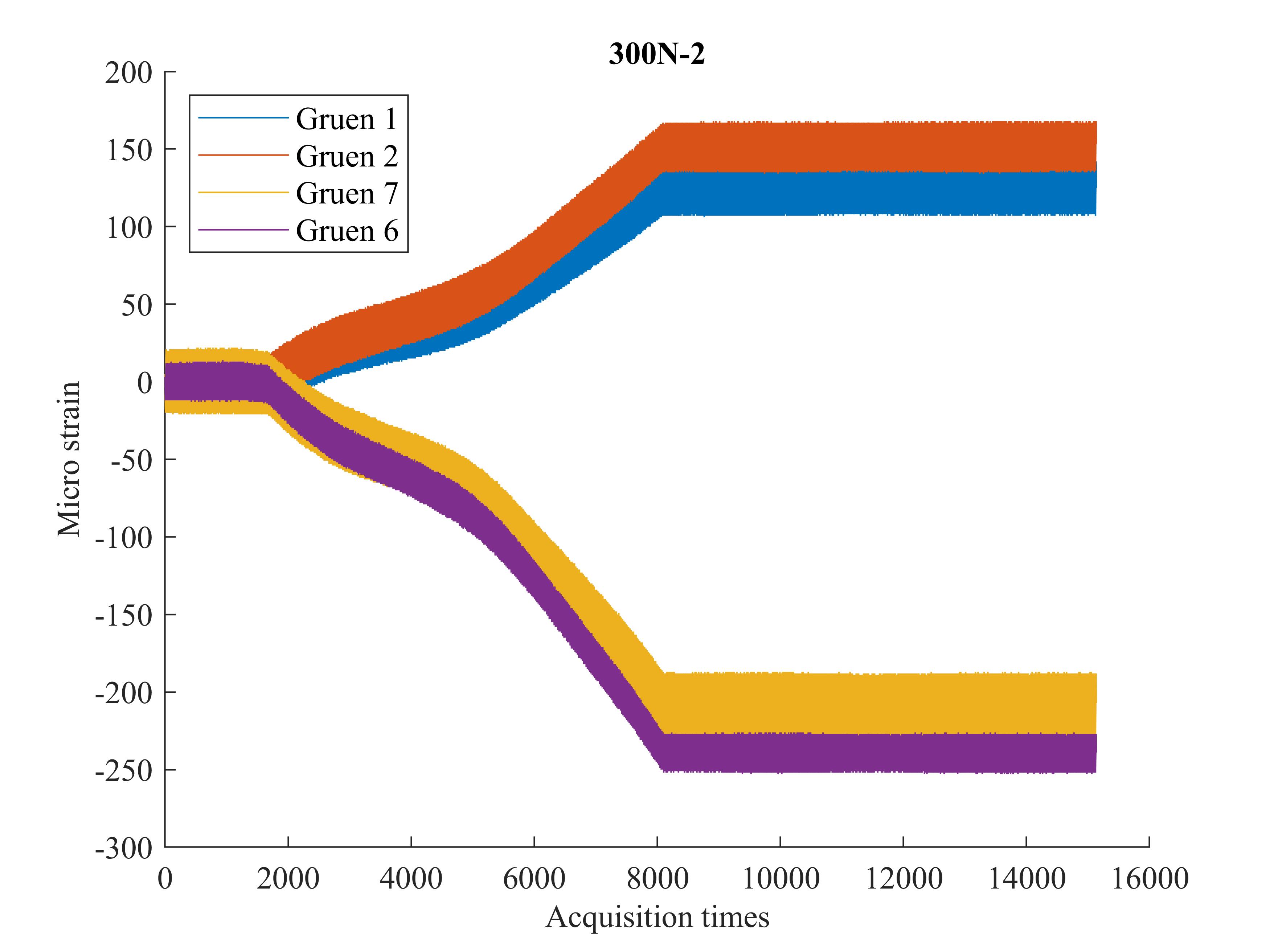

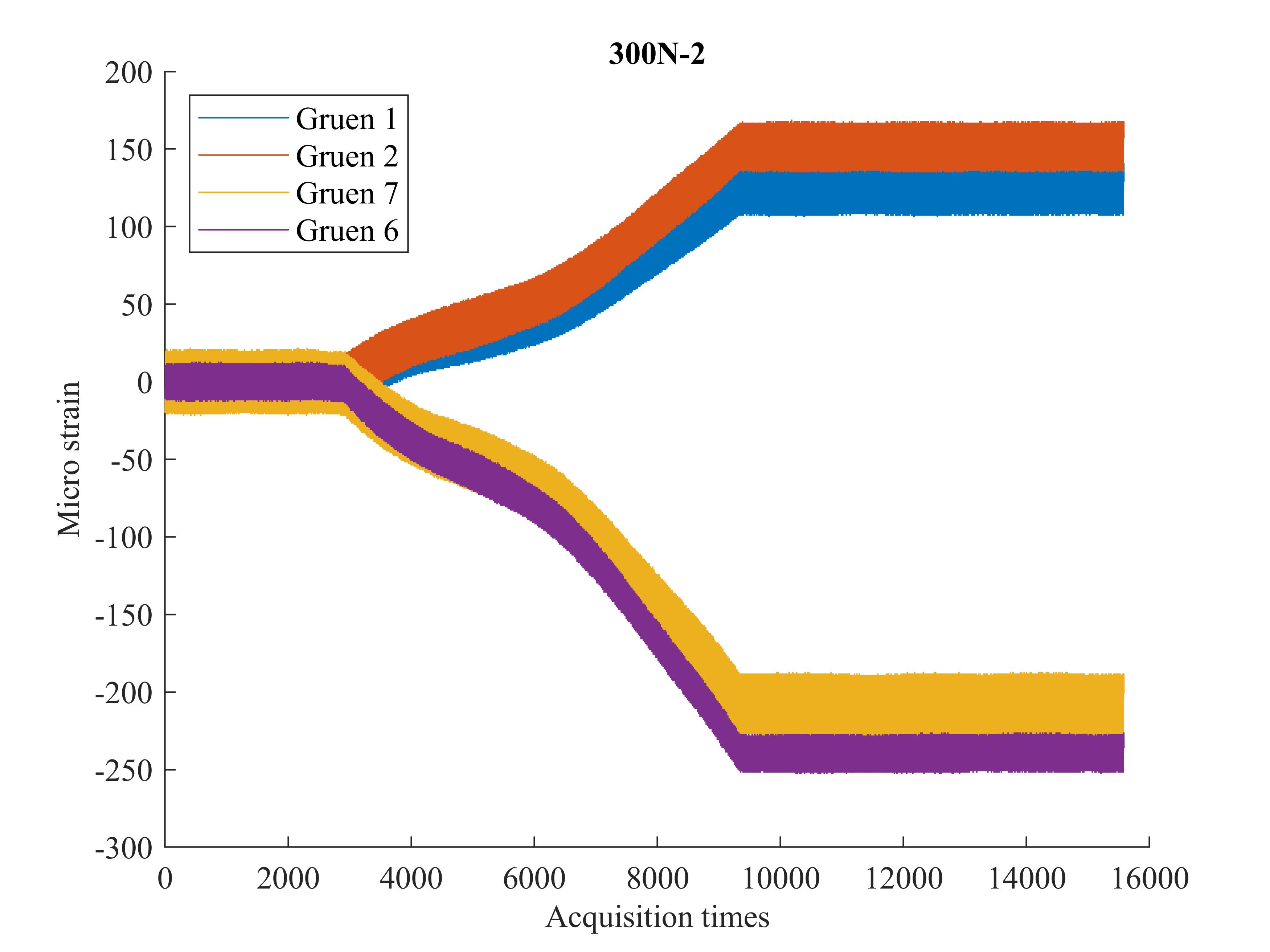


(a) 300N


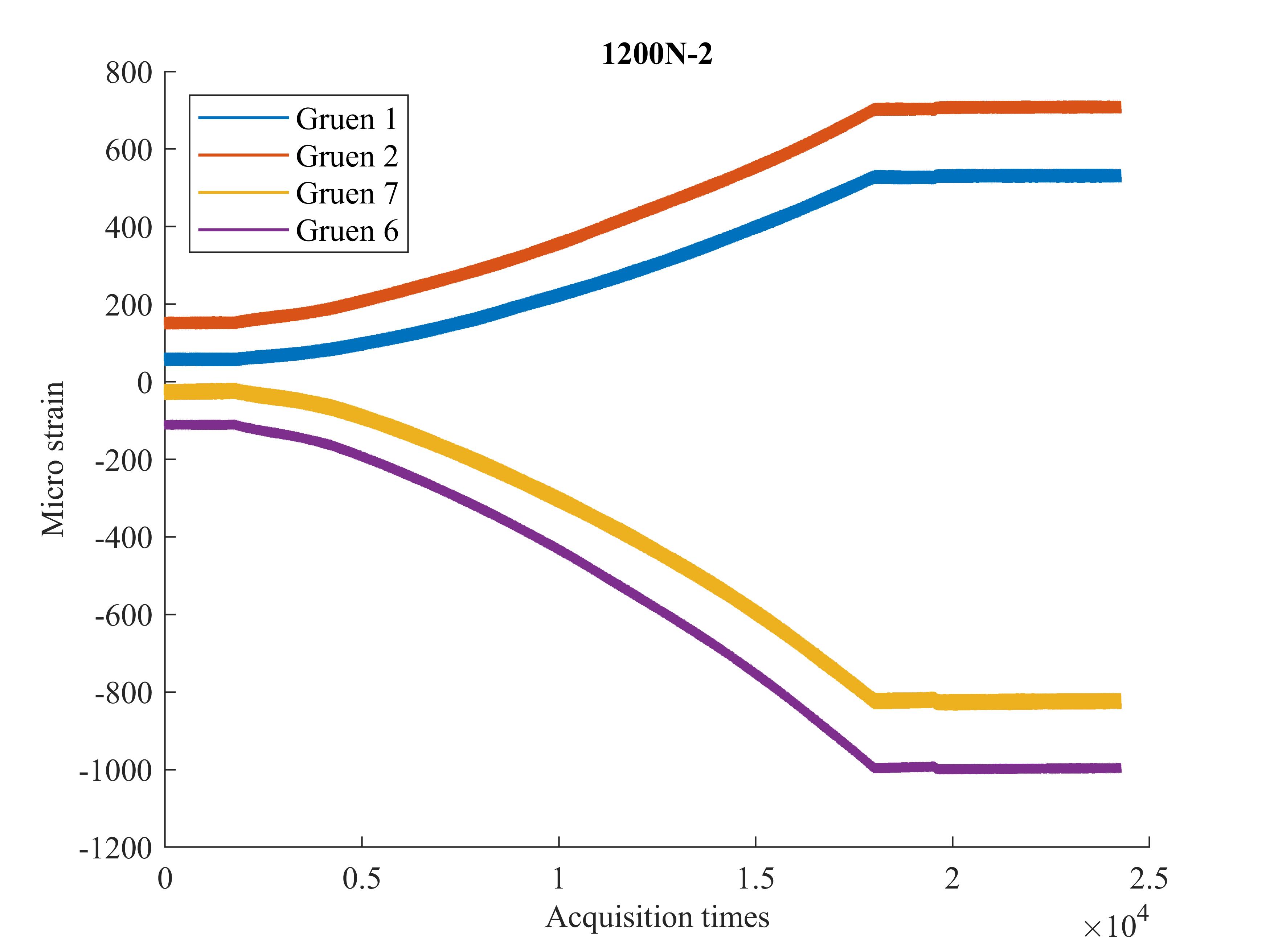

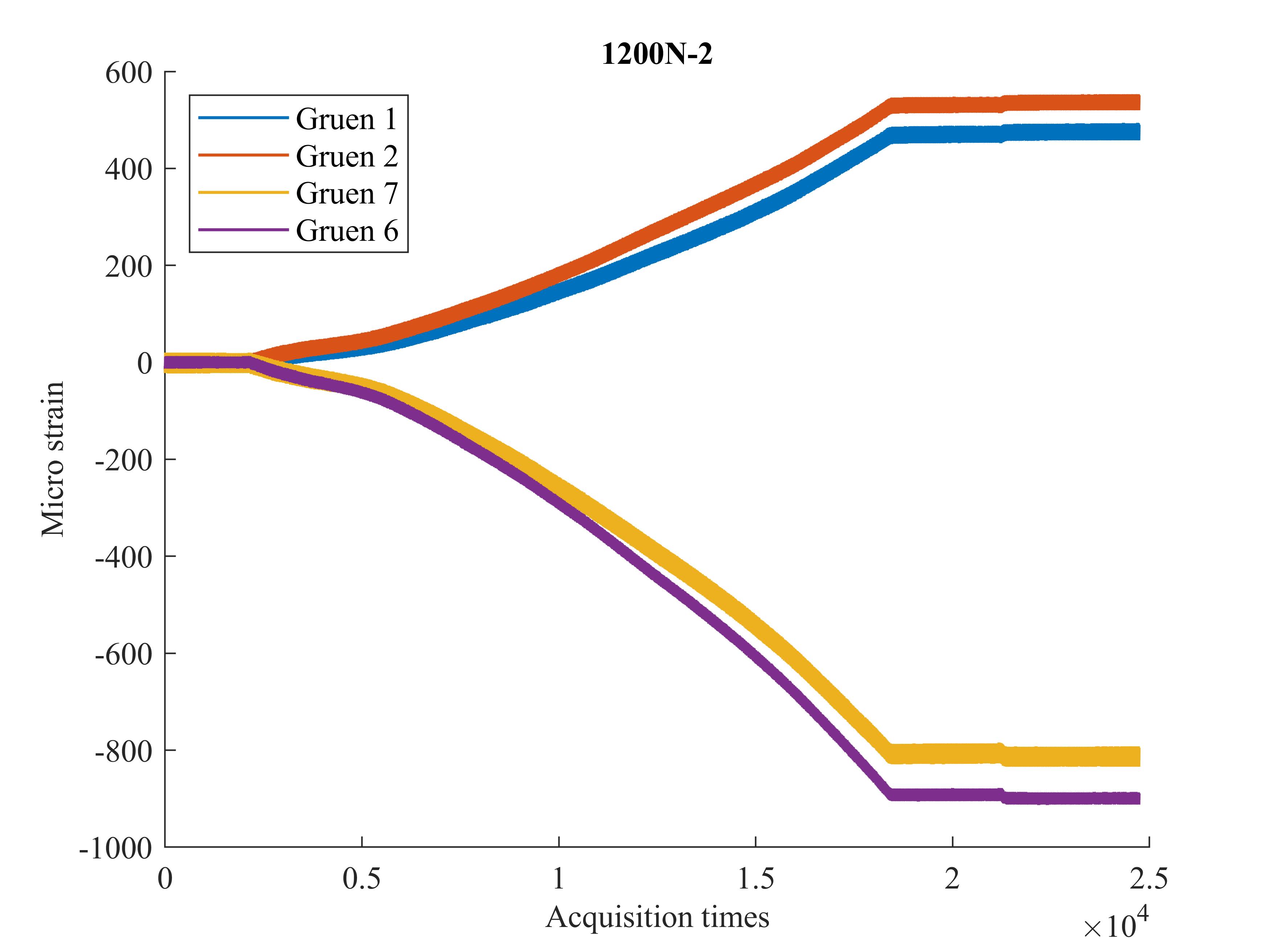

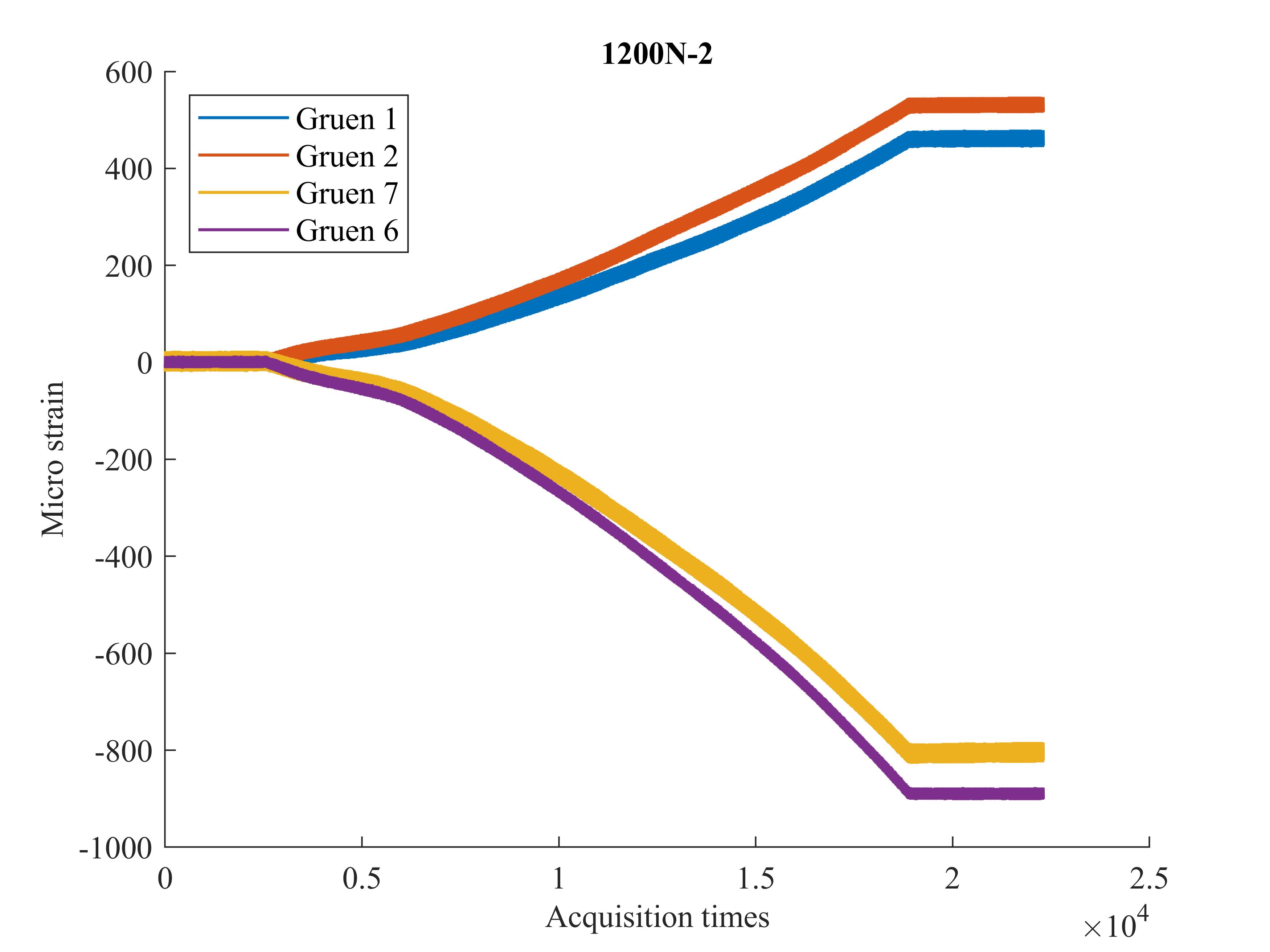

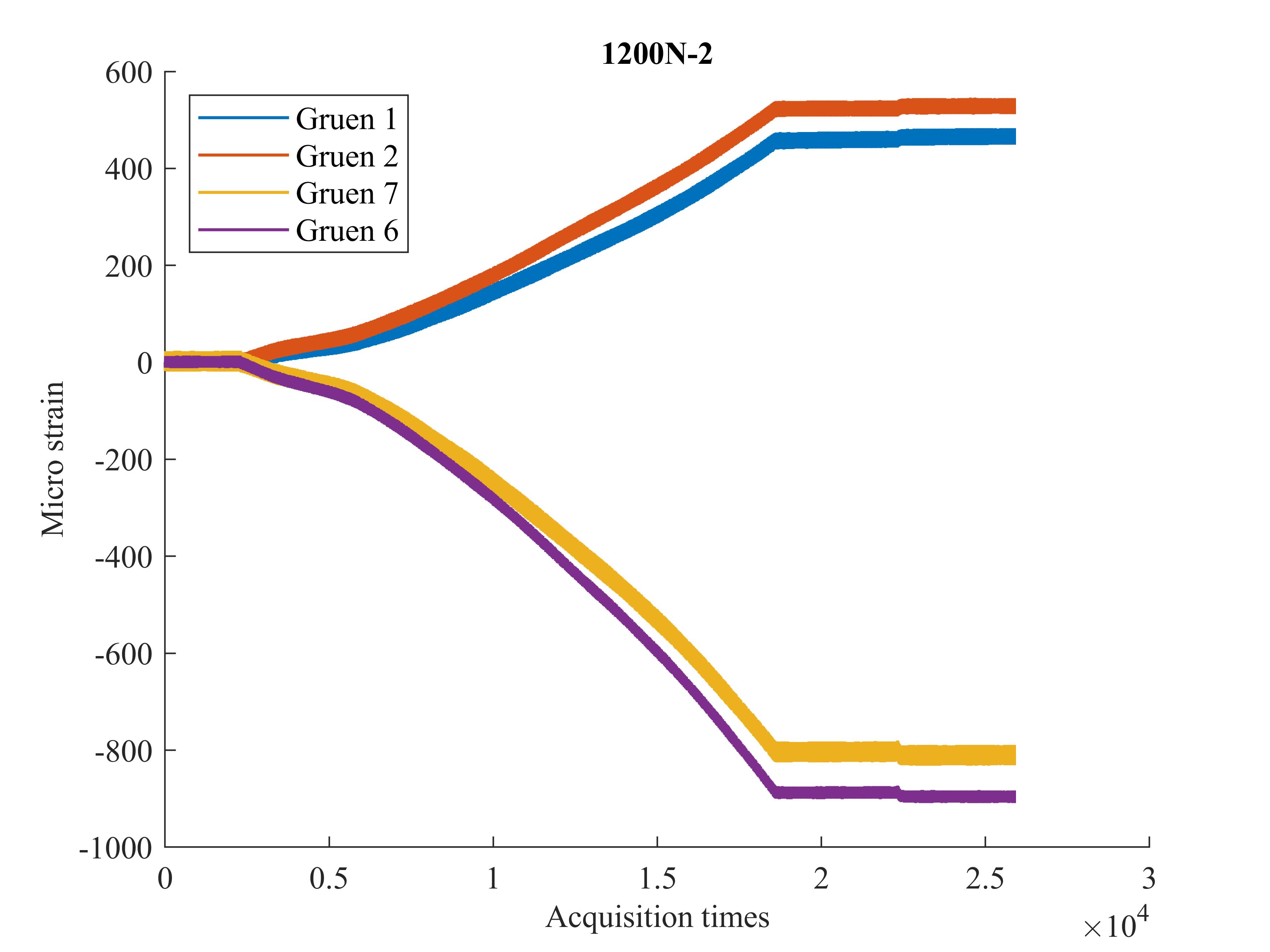

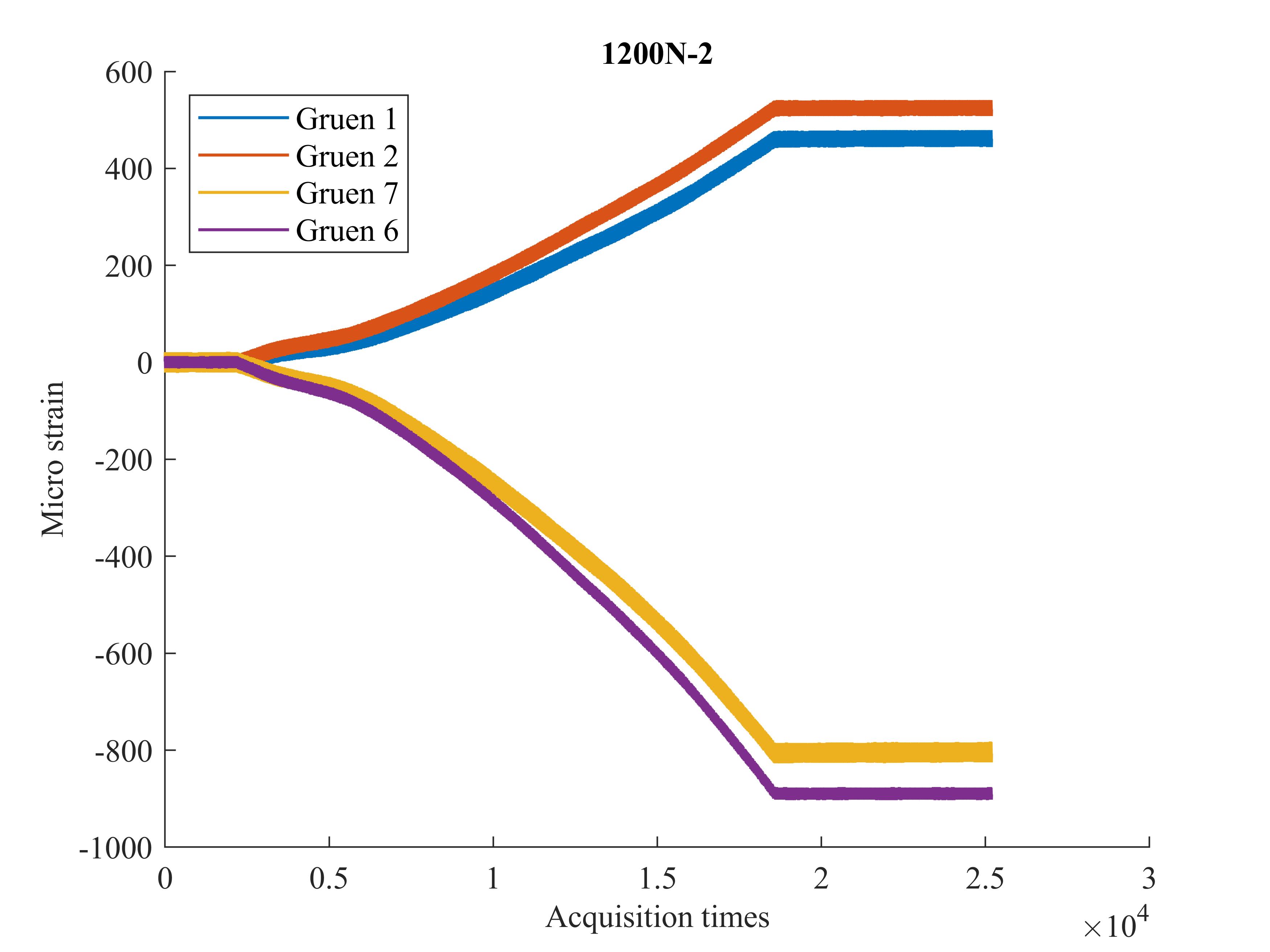

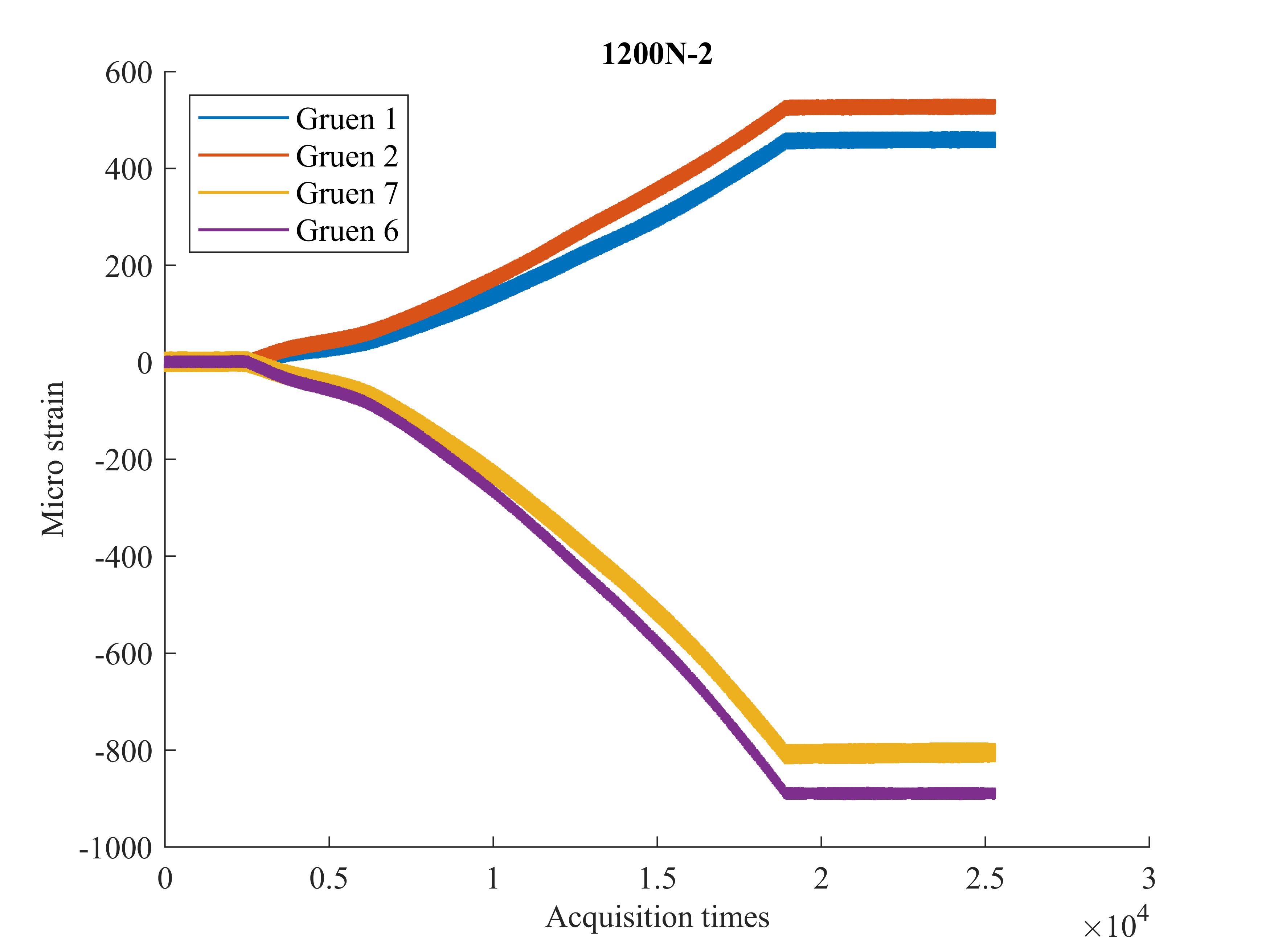

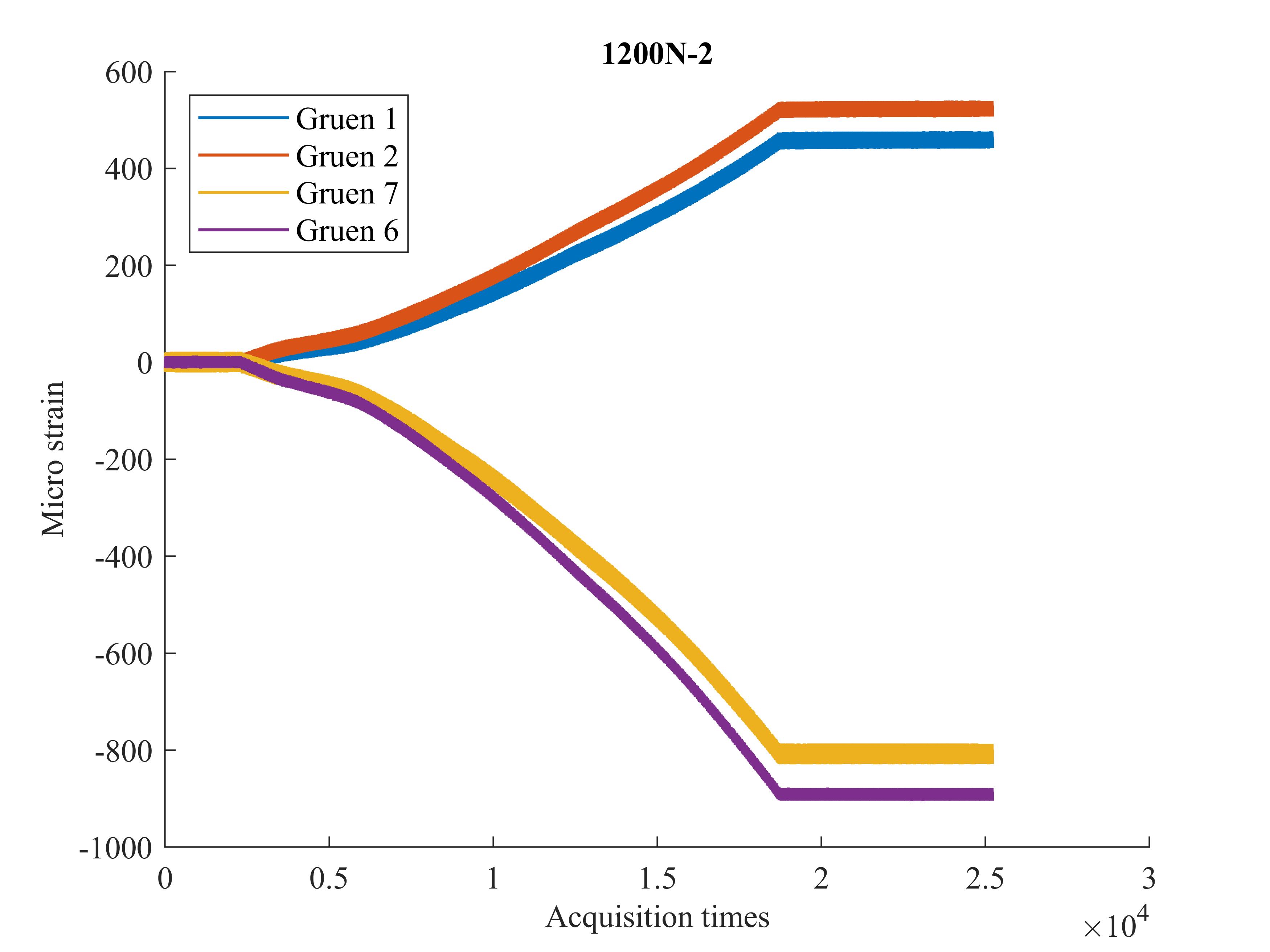

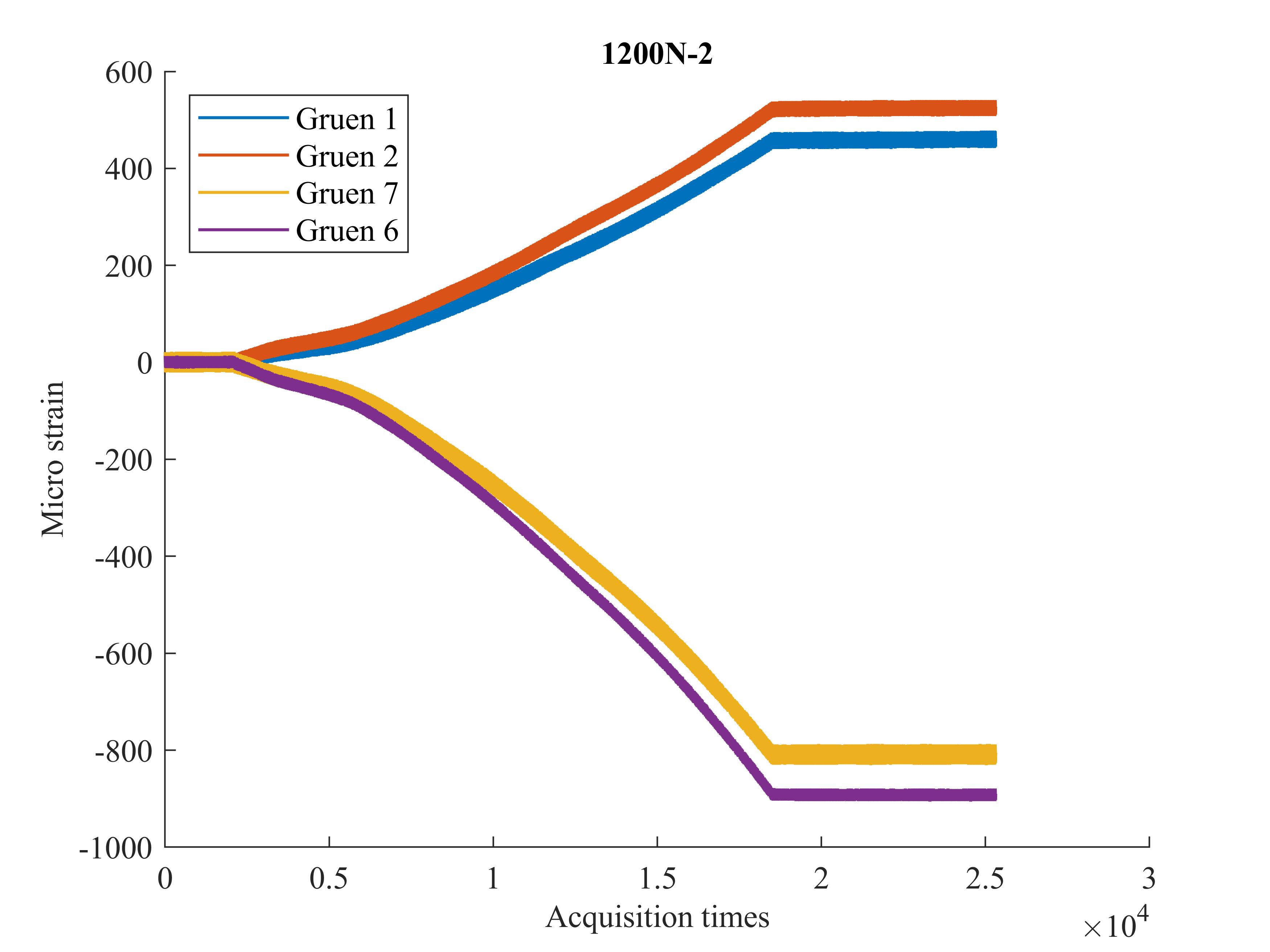

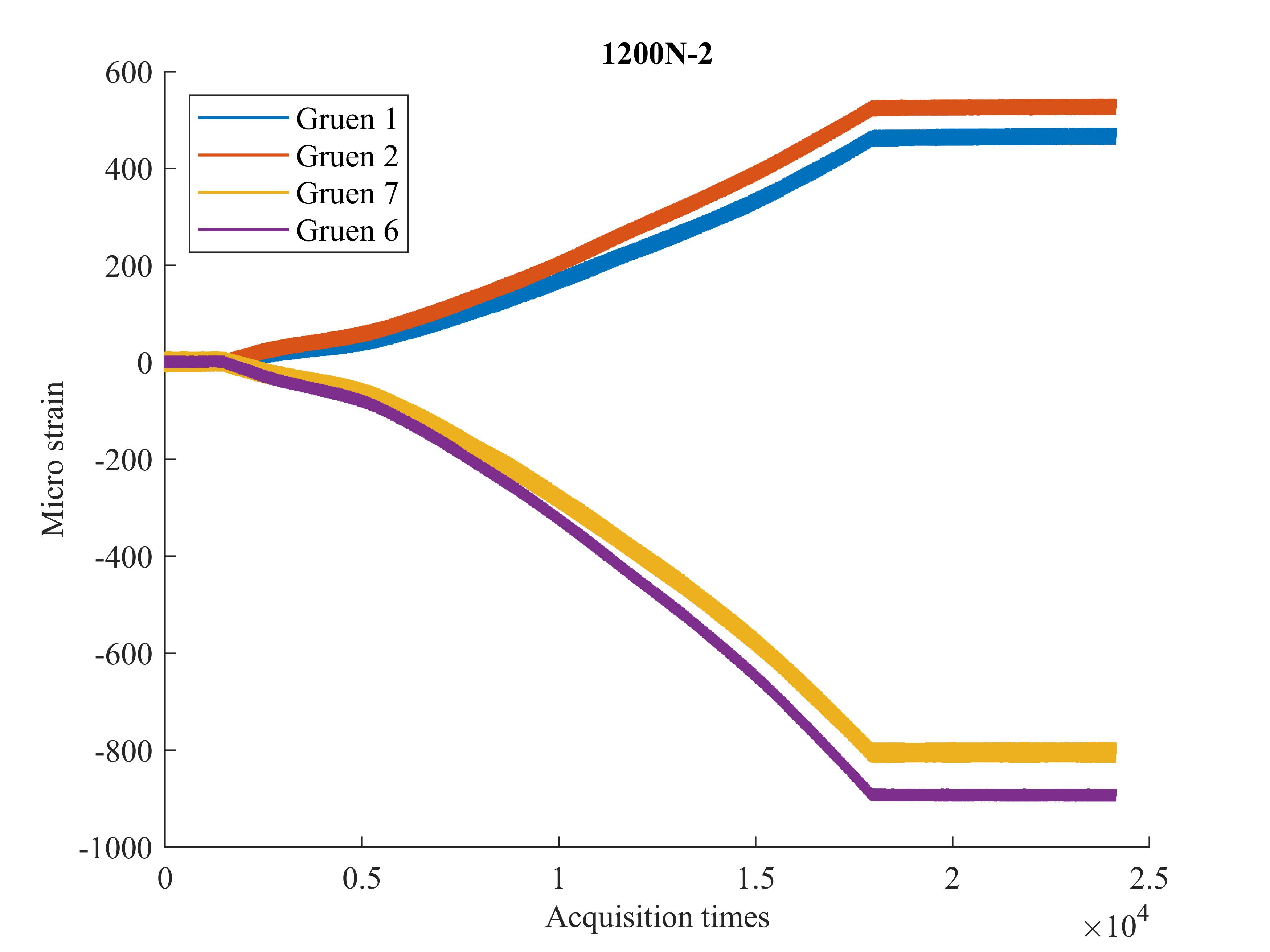

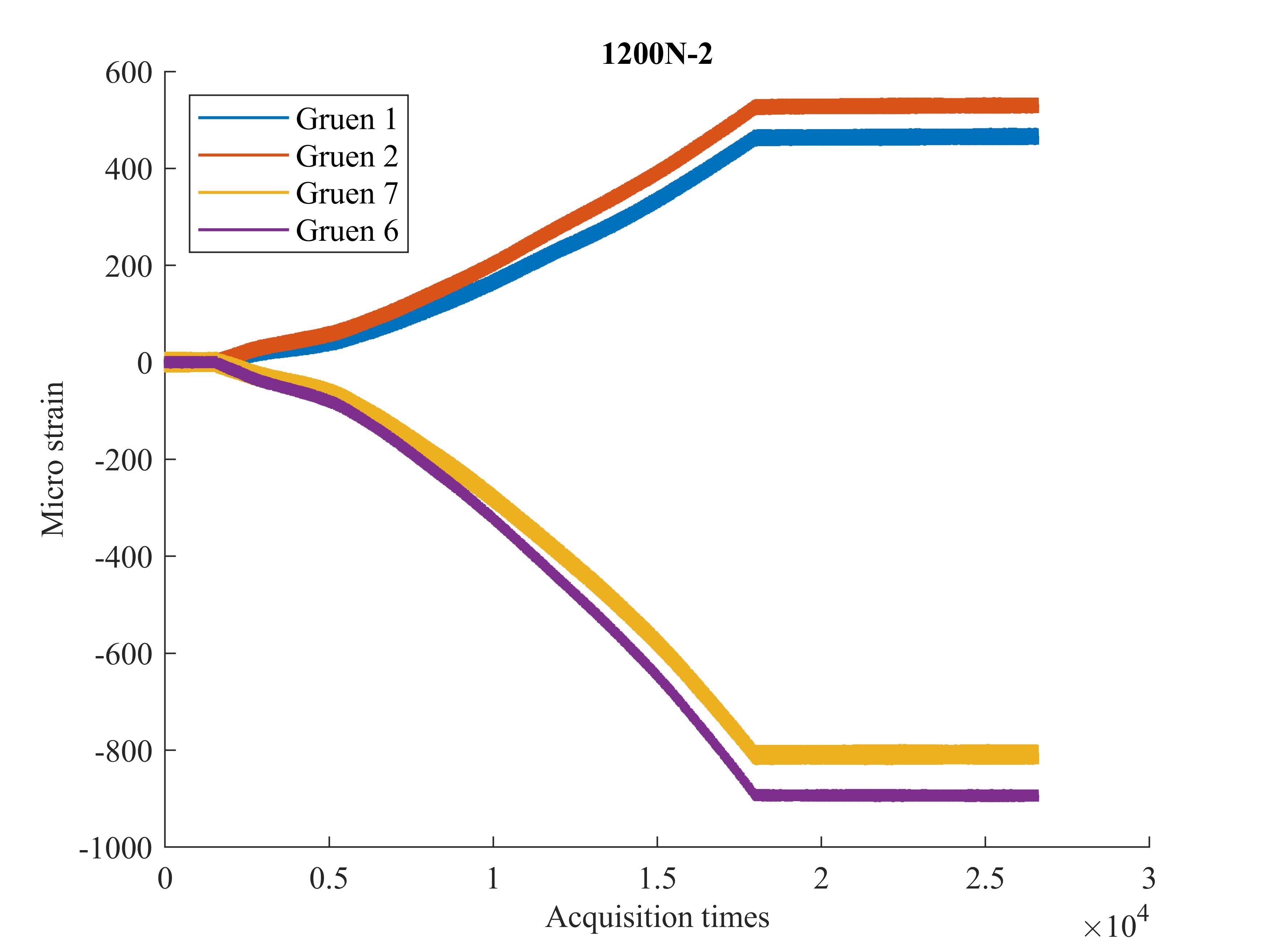


(b)1200N

Fig. S2 Micro-strain values measured in the optimization effect analysis experiment

**Kriging model optimization**

In this study, modern experimental design methods, such as orthogonal design and Latin hypercube design, were used to obtain representative sample points (design schemes), which laid the foundation for developing an effective and accurate approximation model. Subsequently, the correlation function was selected to construct the Kriging approximation model. The Gaussian index model was used as the correlation function, and its expression is given as Eq. S-1.

$R_{k}\left(\theta_{k},x_{k}-x_{k}'\right)=\exp\left(-\theta_{k}\left|\left.x_{k}-x_{k}'\right|\right.^{p_{k}}\right)$ (S-1)

where

**x**, $\text{x}^{'}$ are any two different positions in the design space;

**p** are the model anisotropy parameters, **p*=*** [*p*_1_ *p*_2_ *...p*_m_]^T^;

**θ** are the pending model parameters, $\mathbf{\theta}\text{=}\left[\theta_{1},\theta_{2},...\theta_{m}\right]^{\text{T}}$.

More specifically, **p** determines the smoothness of the approximation model, and $1<p_{k}<2$, *k*=1, 2, …m. In practice, it can be simplified to isotropic (*p_k_*=*p*, *k*=1, 2, ... m), which can provide better robustness. In this study, in order to further improve the accuracy and robustness, the following several measures were adopted.

1. The genetic algorithm (GA) was used to optimize the model parameters **θ** and **p**, and the maximum likelihood estimation was used to maximize the objective function ln*L*, as defined by Eq. S-2.

$\ln L=-\frac{n}{2}\ln\delta^{2}-\frac{1}{2}\ln\left|R\right|$ (S-2)

where

$\delta^{2}=\frac{1}{n}(y_{S}-\beta_{0}F)^{T}R^{-1}(y_{S}-\beta_{0}F)$;

$\beta_{0}=(F^{T}R^{-1}F)^{-1}F^{T}R^{-1}y_{S}$;

$F=\left[\begin{matrix} 1 & 1 & 1 & \cdots& 1 \end{matrix}\right]^{T}$;

*y*_S_ is the output response value;

*R* is the correlation matrix;

*n* is the number of samples.

2. Sample points normalization. The value of each design variable of the sample was transformed into a uniform interval, such as [-1,1] or [0, 1].

3. It was ensured that the correlation matrix **R** in the Kriging model was positive definite. A regularization method can be used; that is, a small constant was added to the diagonal of the matrix **R**, as shown in Eq. S-3.

$R^{'}=R+\text{a}\times I$ (S-3)

where

***I*** is an identity matrix with the same number of rows and columns as **R**;

**a** is a constant, $\text{a}=(10^{3}+n)\varepsilon$, ***ε*** is about 2.22×10^-16^.

**Orthogonal experimental design table (Training samples)**

| Groups | *L*_1_ (mm) | *L*_2_ (mm) | *T* (mm) | *D* (mm) |
| --- | --- | --- | --- | --- |
| 1 | 1.5 | 0.9 | 0.2 | 0.6 |
| 2 | 1.5 | 1 | 0.25 | 0.7 |
| 3 | 1.5 | 1.1 | 0.3 | 0.8 |
| 4 | 1.5 | 1.2 | 0.35 | 0.9 |
| 5 | 1.6 | 0.9 | 0.25 | 0.8 |
| 6 | 1.6 | 1 | 0.2 | 0.9 |
| 7 | 1.6 | 1.1 | 0.35 | 0.6 |
| 8 | 1.6 | 1.2 | 0.3 | 0.7 |
| 9 | 1.7 | 0.9 | 0.3 | 0.9 |
| 10 | 1.7 | 1 | 0.35 | 0.8 |
| 11 | 1.7 | 1.1 | 0.2 | 0.7 |
| 12 | 1.7 | 1.2 | 0.25 | 0.6 |
| 13 | 1.8 | 0.9 | 0.35 | 0.7 |
| 14 | 1.8 | 1 | 0.3 | 0.6 |
| 15 | 1.8 | 1.1 | 0.25 | 0.9 |
| 16 | 1.8 | 1.2 | 0.2 | 0.8 |

**Latin hypercube experimental design table (Training samples)**

| Groups | *L*_1_ (mm) | *L*_2_ (mm) | *T* (mm) | *D* (mm) | Deformation (mm) |
| --- | --- | --- | --- | --- | --- |
| 1 | 1.65 | 0.938 | 0.284 | 0.536 | 0.007194371 |
| 2 | 1.73 | 0.926 | 0.34 | 0.896 | 0.004412291 |
| 3 | 1.41 | 0.854 | 0.3 | 0.992 | 0.004966588 |
| 4 | 1.69 | 0.914 | 0.388 | 0.824 | 0.004604078 |
| 5 | 1.45 | 1.034 | 0.236 | 0.656 | 0.006032078 |
| 6 | 1.87 | 1.058 | 0.324 | 0.632 | 0.00425226 |
| 7 | 1.67 | 1.046 | 0.348 | 0.92 | 0.003507006 |
| 8 | 1.85 | 0.95 | 0.252 | 0.848 | 0.004810379 |
| 9 | 1.55 | 1.01 | 0.356 | 0.68 | 0.00483258 |
| 10 | 1.51 | 1.094 | 0.244 | 1.088 | 0.003280849 |
| 11 | 1.59 | 0.818 | 0.22 | 0.512 | 0.011379451 |
| 12 | 1.49 | 0.962 | 0.38 | 0.776 | 0.004781479 |
| 13 | 1.63 | 0.89 | 0.372 | 0.704 | 0.005762455 |
| 14 | 1.75 | 0.83 | 0.276 | 1.016 | 0.004606582 |
| 15 | 1.57 | 1.07 | 0.396 | 0.608 | 0.004197012 |
| 16 | 1.81 | 0.902 | 0.268 | 0.944 | 0.004689569 |
| 17 | 1.79 | 1.082 | 0.26 | 0.752 | 0.004120761 |
| 18 | 1.71 | 0.842 | 0.308 | 1.04 | 0.004184491 |
| 19 | 1.47 | 0.866 | 0.212 | 0.728 | 0.008140463 |
| 20 | 1.43 | 0.878 | 0.332 | 1.064 | 0.00401546 |
| 21 | 1.61 | 1.022 | 0.316 | 0.872 | 0.004089643 |
| 22 | 1.83 | 0.806 | 0.364 | 0.56 | 0.007603169 |
| 23 | 1.89 | 0.974 | 0.292 | 0.968 | 0.003780041 |
| 24 | 1.53 | 0.998 | 0.228 | 0.584 | 0.007018256 |
| 25 | 1.77 | 0.986 | 0.204 | 0.8 | 0.005294972 |
